# Next-Generation Bioplastics for Food Packaging: Sustainable Materials and Applications

**DOI:** 10.3390/ma18122919

**Published:** 2025-06-19

**Authors:** Xiaokun Shi, Lijuan Cui, Chao Xu, Shuping Wu

**Affiliations:** Institute of Polymer Materials, School of Materials Science & Engineering, Jiangsu University, Zhenjiang 212013, China; 2212405057@stmail.ujs.edu.cn (X.S.); 2222305061@stmail.ujs.edu.cn (L.C.); 2212205008@stmail.ujs.edu.cn (C.X.)

**Keywords:** bioplastics, food packaging, processing technology, sustainable materials

## Abstract

As the global plastic pollution problem intensifies and the environmental hazards of traditional petroleum-based plastics become increasingly significant, the development of sustainable alternative materials has become an urgent need. This paper systematically reviews the research progress, application status and future trends of new generation bioplastics in the field of food packaging. Bioplastics are categorized into three main groups according to their sources and degradability: biobased biodegradable materials (e.g., polylactic acid PLA, polyhydroxy fatty acid ester PHA, chitosan, and cellulose-based materials); biobased non-biodegradable materials (e.g., Bio-PE, Bio-PET); and non-biobased biodegradable materials (e.g., PBAT, PCL, PBS). Different processing technologies, such as thermoforming, injection molding, extrusion molding and coating technologies, can optimize the mechanical properties, barrier properties and freshness retention of bioplastics and promote their application in scenarios such as food containers, films and smart packaging. Although bioplastics still face challenges in terms of cost, degradation conditions and industrial support, promising future directions are found in the development of the large-scale utilization of non-food raw materials (e.g., agricultural waste, algae), nano-composite technology to enhance the performance, and the development of intelligent packaging functions. Through technological innovation and industry chain integration, bioplastics are expected to transform from an environmentally friendly alternative to a mainstream packaging material, helping to realize the goal of global carbon neutrality.

## 1. Introduction

Plastics are synthetic materials composed of long-chain polymer molecules that are used in a wide range of applications, such as packaging, construction, automotive, and medical care, because they are lightweight, durable, and cost effective [1,2,3,4,5]. Global plastics production has grown exponentially since the mid-20th century. By 2050, plastics and other petrochemicals are projected to account for nearly half of the incremental demand for oil and to consume 15 percent of the world’s carbon budget [6,7,8]. However, the widespread use of plastics has also brought about environmental pollution problems, such as the accumulation of plastic waste in the oceans and on land, as well as the consumption of resources and greenhouse gas emissions during the production process, which can lead to serious pollution of marine and terrestrial ecosystems, among others [9,10,11,12,13].

The food packaging sector is undergoing a profound transformation as the problem of plastic pollution intensifies through globalization. As the leading area of plastics consumption, traditional petroleum-based plastic packaging materials have been criticized for their long-term residual nature, posing not only a threat to biodiversity, but also a potential risk to human health through the food chain, due to their long-term residual nature [14,15,16,17,18]. Against this backdrop, bioplastics, with their renewability and environmental friendliness, have become a strategic choice to replace traditional plastics. This transition not only reduces the environmental impact of packaging materials but also ensures food safety through material innovation [19,20,21].

**Figure 1:**
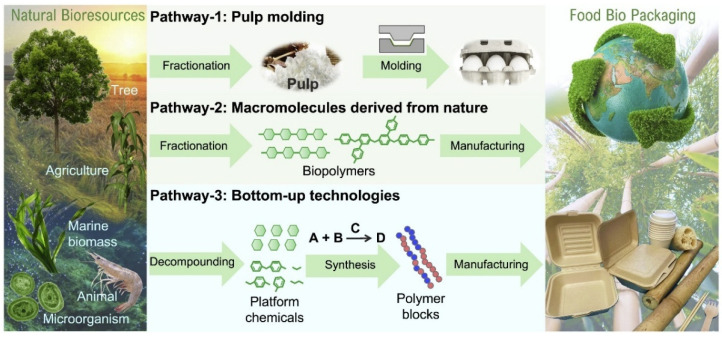
Schematic diagram of three pathways for the rational design of biopackaging from natural resources to food. Reprinted from [32] with permission from Elsevier.

Bioplastics are a new family of materials derived from natural, renewable resources, such as agricultural crops, herbaceous fibers and marine organisms [22,23,24]. Figure 1 shows three ways to rationalize the design of bioplastics for food packaging from natural resources, valued for their biodegradability and environmental friendliness. As defined by the European Bioplastics Association, such materials can be categorized as bio-based, biodegradable or both [25]. “Biobased” materials are partially or entirely made of biomass instead of fossil resources, while ‘biodegradable’ materials can be broken down by microorganisms into carbon dioxide and water [26,27,28,29,30,31]. This dual property enables them to demonstrate unique advantages in the food packaging sector: they reduce dependence on petroleum resources and reduce environmental pressure through natural recycling.

**Figure 2:**
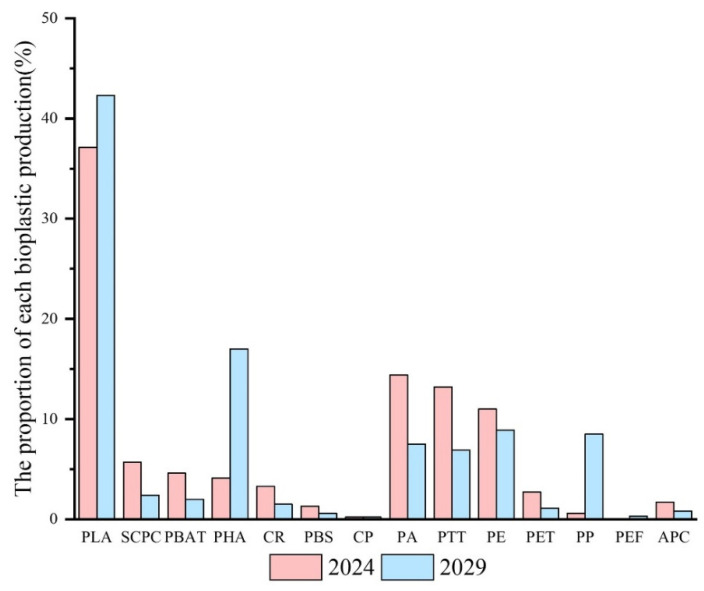
Global production capacities of bioplastics in 2024 and 2029 [33].

Bioplastic, because of its degradability and renewable resources, has become an ideal candidate for replacing traditional plastics. Bioplastics currently account for about half of the annual production of plastics; the total global production of plastics continues to grow steadily, and the global production capacity of bioplastics is projected to rise significantly from approximately 2.47 million tons in 2024 to around 5.73 million tons by 2029 [33]. Figure 2 illustrates the growth trajectory of the global bioplastics production capacity between these two years. This upward trend reflects the expanding role of bioplastics in addressing sustainability challenges and meeting increasing demand across various industries. According to [33], polylactic acid (PLA) [34,35], polyhydroxy fatty acid esters (PHAs), and starch-based materials [36,37,38] exhibit strong mechanical properties, thermal stability, and good oxygen and moisture barrier capabilities, giving them the potential for a wide range of applications in food packaging [39,40,41]. In addition, by combining bioplastics with active additives, it is possible not only to enhance the antimicrobial properties of bioplastics but also to achieve a higher quality of protection for foodstuffs [42,43].

Bioplastics also have several drawbacks in practical applications. First, their production costs are higher than those of traditional plastics, limiting their widespread adoption. Second, they have performance limitations, such as low mechanical strength, poor barrier properties, and insufficient thermal stability. For example, pure PLA is highly brittle, cellulose-based films have high oxygen permeability, and PHA has poor heat resistance. Third, their degradation requires specific conditions, and they degrade slowly in natural environments. For example, PBAT may take several months to years to fully degrade in natural soil. Fourth, the recycling system for bioplastics also has some shortcomings. They are often difficult to distinguish from traditional plastics, and immature chemical recycling technologies further complicate recycling efforts. Additionally, their raw materials sometimes rely on crops such as corn, posing a risk of competing with food crops for land. These drawbacks all hinder the widespread adoption of bioplastics [44,45,46,47]. In recent years, researchers have significantly improved the thermal, mechanical and barrier properties of bioplastics through nano-enhancement composite design and other technological means, laying a technological foundation for their practical application [48,49,50,51].

This review systematically compiles the research progress of bioplastics, focusing on analyzing their material properties, production processes and suitability in food packaging. By exploring the sustainable development path of bioplastics and revealing their key role in realizing the transformation of packaging materials into low-carbon and harmless materials, this research provides theoretical support and technological outlook for promoting the green revolution in the food packaging industry.

## 2. Classification and Characterization of Bioplastics

The definition and classification system of bioplastics, as a new type of material with a wide range of applications, is of great significance in understanding their environmental value and technical properties. According to the European definition of bioplastics, bioplastics refer to a broad family of materials that are biobased, biodegradable or both [25]. From the dual dimensions of material source and degradation characteristics, bioplastics can be systematically divided into three major categories, with significant differences in environmental benefits and technical characteristics. First, bio-based biodegradable materials constitute the most ecologically valuable category, with raw materials primarily derived from natural polymers, such as polysaccharides (starch, cellulose, chitin, etc.) and proteins [30,52,53], as well as biosynthetic polyester materials, such as polylactic acid (PLA) and polyhydroxyalkanoates (PHAs) [54]. These materials not only realize renewable raw materials, but their biodegradable characteristics effectively solve the white pollution problem of traditional plastics, thus, showing significant advantages in the fields of food packaging, agricultural films, etc. This is in contrast to biodegradable materials, the second category of bio-based non-degradable plastics. Although these materials also use bio-based raw materials, such as bio-based polyethylene Bio-PE, bio-based polyethylene terephthalate Bio-PET, etc., their chemical structures are homologous to traditional petroleum-based plastics [55]. This isomorphism gives them excellent mechanical strength and thermal stability and enables them to be directly adapted to existing plastics processing equipment; however, there are significant limitations in terms of environmental sustainability. Due to the lack of biodegradability, they still need to rely on physical recovery systems for recycling. Furthermore, degradability is not an exclusive property of bio-based materials. The breakthrough development of the third category of fossil-based degradable plastics, such as PBAT, PCL, PBS, etc., reveals the separability of degradability from the source of the feedstock [26]. The materials are derived from petroleum resources but are designed to be biodegradable. Although these materials are derived from petroleum resources, they realize controlled biodegradation through molecular structure design, and show unique application value in specific application scenarios (e.g., demanding industrial composting environments). The existence of such technological pathways not only broadens the technological boundaries of bioplastics but also prompts the academic community to re-examine the multidimensional evaluation criteria of material sustainability.

### 2.1. Biobased Biodegradable Bioplastics

#### 2.1.1. PLA

**Figure 3:**
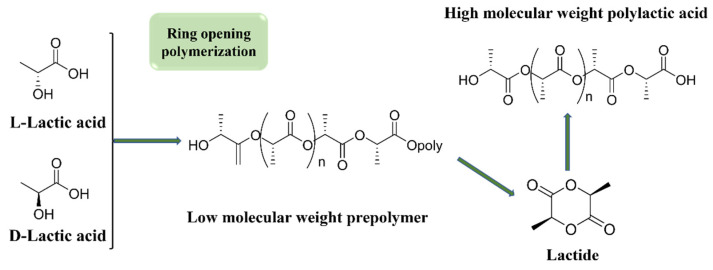
Synthesis of polylactic acid produced by ring-opening polymerization.

As a third-generation bio-based biodegradable material, polylactic acid (PLA) is usually prepared industrially using a “two-step” process: firstly, L-lactic acid monomer is produced by fermentation of corn starch and other renewable resources, and then propylenic ester intermediates are generated by cyclization, and ultimately high molecular weight PLA can be obtained by ring-opening polymerization [56,57,58,59]. Figure 3 illustrates the process of producing polylactic acid (PLA) through ring-opening polymerization. PLA is biocompatible and biodegradable (ultimately degrading completely into carbon dioxide and water), but its complete degradation depends on specific industrial composting conditions, including sustained high temperatures, high humidity (to meet the conditions for hydrolysis reactions), specific microbial communities, and a neutral to slightly alkaline pH value. If these conditions are not met, PLA degradation in natural environments will be extremely slow or even stagnant. In addition, PLA has outstanding thermal stability, transparency, and processing performance, making it widely used in food packaging, medical supplies, and other fields [60,61,62,63,64,65]. However, its industrialization is constrained by defects such as intrinsic brittleness, high gas permeability, and high production cost, which have prompted researchers to break through the performance bottleneck through various modification techniques.

In food packaging, the antioxidant effect of PLA is comparable to that of traditional plastics (e.g., PET, PE), and there is no migration of harmful substances [66]. However, to solve the deficiencies of its mechanical and barrier properties, nanocomposite modification technology has shown significant advantages. For example, the *E. coli* bacterial inhibition rate can reach 99.9% by TiO_2_/graphene oxide blend system (1.5 wt.% addition), while the PLA coating modified by olive leaf extract has broad-spectrum antimicrobial properties (inhibition rate of *Staphylococcus aureus* > 95%), but there is a technical bottleneck of migrants exceeding the standard of EU 10/2011 (exceeding the standard of EU 10/2011 by 20%) [67,68]. In addition, although PLA possesses biocompatibility and degradability, it suffers from high brittleness, high permeability and high cost; its performance can be optimized by starch-embedded modification, but the abundant source of starch also has defects. Godoy et al. first extracted starch from avocado seeds with a yield of 20.31 ± 0.5% after washing, lyophilization, and alkali treatment, with a composition containing 13.045 ± 0.847% humidity, 3.449 ± 0.273% protein, etc., and a content of straight-chain starch of 29.651 ± 0.986%. Subsequently, m-St with different molar ratios (1:2, 1:4, 1:6) was prepared in DMSO medium using t-BAA as a modifier. The success of the modification was confirmed through characterization techniques such as FTIR, NMR, etc., and the modified starch showed enhanced hydrophobicity, altered thermal stability and glass transition temperature, and increased molecular weight. Next, the composites were made by melt blending and hot pressing m-St with PLA at 15 wt.% and 20 wt.%, and PLA/N-St composites were used as a control. The thermal property tests showed that the thermal stability of the composites decreased with the increase in m-St ratio and content; the microstructure showed that m-St improved the interfacial compatibility with PLA; in terms of the mechanical properties, the tensile strength and elastic modulus decreased in most of the PLA/m-St composites, but the elongation at break was increased from 3.35% to 27.80% when adding 20 wt.% m-St (1:6). The UV barrier capability of PLA/m-St composites is significantly enhanced, and their water vapor and oxygen barrier properties are greatly improved, approaching the ideal index for perishable food packaging, and outperforming pure PLA and common petroleum-based plastics. In this study, PLA/m-St composites with good tensile strength, high elongation at break, and excellent UV blocking and barrier properties were prepared for the first time [69].

**Figure 4:**
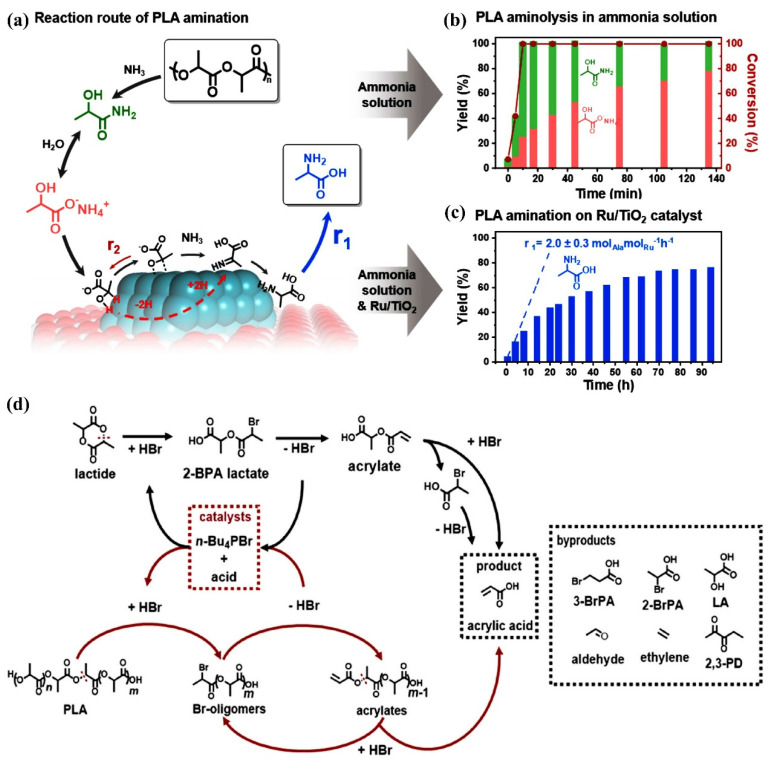
Catalytic transformation of PLA to alanine. (**a**) Proposed reaction mechanism of PLA amination on a Ru/TiO_2_ catalyst in ammonia solution. (**b**) Aminolysis of PLA in NH_3_·H_2_O without a catalyst. (**c**) Amination of PLA with 2 wt.% Ru/TiO_2_. Conditions: PLA 1.0 g, metal/PLA monomer molar ratio 0.024, 50 mL of NH_3_·H_2_O (25 wt.%), N_2_, 140 °C. Reprinted from [70] with permission from American Chemical Society. Catalytic cracking of PLA to acrylic acid. (**d**) Proposed reaction routes of lactide/PLA cracking with acid catalysts in ionic liquid. Reprinted from [71] with permission from John Wiley and Sons.

Significant advancements have been achieved in the recycling technology of polylactic acid (PLA), as depicted in Figure 4. This figure illustrates the approximate process of catalytic conversion of PLA into alanine. This innovative approach not only enhances the sustainability of PLA by enabling its efficient recycling but also highlights the potential for transforming waste materials into valuable chemical products. (1) Converting plastic waste into high-value-added chemicals is also an important direction in bioplastics research. Although PLA degrades slowly in the natural environment and emits carbon dioxide, natural degradation is often not its ideal disposal method. Instead, the conversion of PLA into valuable chemicals, such as alanine, has emerged. Tian et al. converted PLA into alanine by an innovative one-pot catalytic method in the presence of a Ru/TiO_2_ catalyst and ammonia solution at 140 °C without the need to add additional hydrogen. Moreover, after screening several catalysts, Ru/TiO_2_ was found to be the most effective with good stability. The reaction started from the fifth cycle, which was converted to PLA mixed with recovered ammonium lactate and lactam ide, with the total selectivity of alanine reaching 94% and the purity reaching 95% after eight cycles; the accuracy of this experiment was verified by pipetting, which gave 3.0 g of purified alanine from 5.0 g of pipette after five cycles. All these innovative recycling methods open up new, efficient and environmentally friendly paths for PLA recycling [70]. (2) Jiao et al. formed oligomers containing Br or acryloyl groups by attacking PLA using Br^−^ and H^+^, which were then interconverted to produce acrylic acid, which was collected by distillation. During the study, the addition of Pure Terephthalic Acid (PTA) was found to enhance the acrylic acid yield and shorten the reaction time, and the effects of different substrates, acid catalysts and ionic liquids were also examined. After optimizing the conditions, cyclic experiments were carried out with commercial PLA pipettes, and finally, an amount of high-purity acrylic acid was obtained. The process achieved an efficient conversion of PLA to acrylic acid under mild conditions, culminating in about 1.7 g of acrylic acid in an ethanol solution in the trap, and 1.2 g of acrylic acid (purity > 95%) was collected after purification. Its high atom economy provides a new idea for the upcycling of plastic waste and promotes the application of ionic liquids in catalytic reactions [71]. (3) Murtz et al. focused on electrochemical depolymerization recovery of polylactic acid (PLA). There are few studies on electrochemical recovery of PLA due to the shortcomings of conventional recovery methods. The study was conducted using dioxane and water (9:1) as the medium and lithium perchlorate as the electrolyte and experimented with platinum electrodes. The results showed that the maximum yield of lactic acid was 87% at a current density of 50 mA cm^−2^ and a charge of 1000 C. It is hypothesized that the reaction contains single electron transfer and water electrolysis nucleophilic attack pathways, which are stochastic chain breaking. As for the influence of reaction conditions, PLA concentration, current density, electrode material, and polymer chain length all had different degrees of influence on the yield [72]. (4) Enzymatic hydrolysis, on the other hand, has the advantage of reacting under mild conditions and high specificity, and is expected to enable efficient recovery of PLA; however, it currently suffers from high costs and a lack of effective enzyme producers. Myburgh et al. constructed a recombinant brewer’s yeast (Saccharomyces cerevisiae) and caused it to express PLA hydrolase from a fungus. The genes encoding the keratinase-like enzyme (CLE1) and protease K (Pro K) were selected, codon optimized and introduced into Saccharomyces cerevisiae strain Y294. The results showed that codon optimization significantly enhanced the expression and hydrolytic activity of CLE1, with the Y294 CLE ns strain showing the most outstanding performance. The crude supernatant of this strain was able to effectively hydrolyze PLA emulsion powder and film, releasing 9.44 g/L lactic acid from 10 g/L PLA film, which resulted in a weight loss of more than 40% of the film. The hydrolysis mechanism showed that the CLE1 enzyme preferentially acted on the amorphous region of the PLA film, followed by the crystalline region. It was further verified that the crude supernatant of Y294 CLE ns strain had the ability to hydrolyze PLA polymers of different molecular weights [73].

#### 2.1.2. PHA

Polyhydroxyalkanoate (PHA) is a polymer material synthesized by microorganisms with biodegradability, biocompatibility, and gas barrier properties [74,75,76,77,78]. There is a wide range of PHA types, including short- and medium- to long-chain PHAs, as well as their copolymers, such as PHB, PHBV and PHB HHX. The glass transition temperature of short-chain PHA is generally −4.6 to 6.7 °C, depending on the crystallinity in the film [79,80]. However, long-chain PHAs, such as (PHO), due to their special state when present at room temperature, often require special treatment conditions during processing. These materials not only have the thermoplasticity of traditional plastics, but also have unique biological properties that make them widely used in medical, packaging, 3D printing, etc. The biodegradability of PHA makes it an ideal alternative to traditional plastics, which is conducive to helping humans reduce the environmental pollution caused by petroleum-based plastics [81,82,83].

Currently, there are three main routes in PHA production: microbial, enzymatic, and chemical. Microbial fermentation method uses bacteria to synthesize PHA under environmental pressure, involving a variety of metabolic pathways and enzymes, such as PHAA, PHAB, PHAC, etc. Factors such as microbial strains, raw materials, and fermentation conditions affect the yield and quality of PHA, and this method has excellent performance in molecular weight, relatively low production costs, and mature technology for large-scale production. Enzymatic synthesis can better control the polymer properties, but the high cost of high-quality synthases, monomers and auxiliaries, the difficulty in producing monomers, and the inhibitory effect of CoA limit its large-scale application [84,85,86,87,88]. The most commonly used chemical synthesis method is ring-opening polymerization, which can synthesize a variety of polymers, but there are problems such as the high cost of using enantiomerically pure lactone and the susceptibility to ester exchange reactions leading to polymer polydispersity [89].

**Figure 5:**
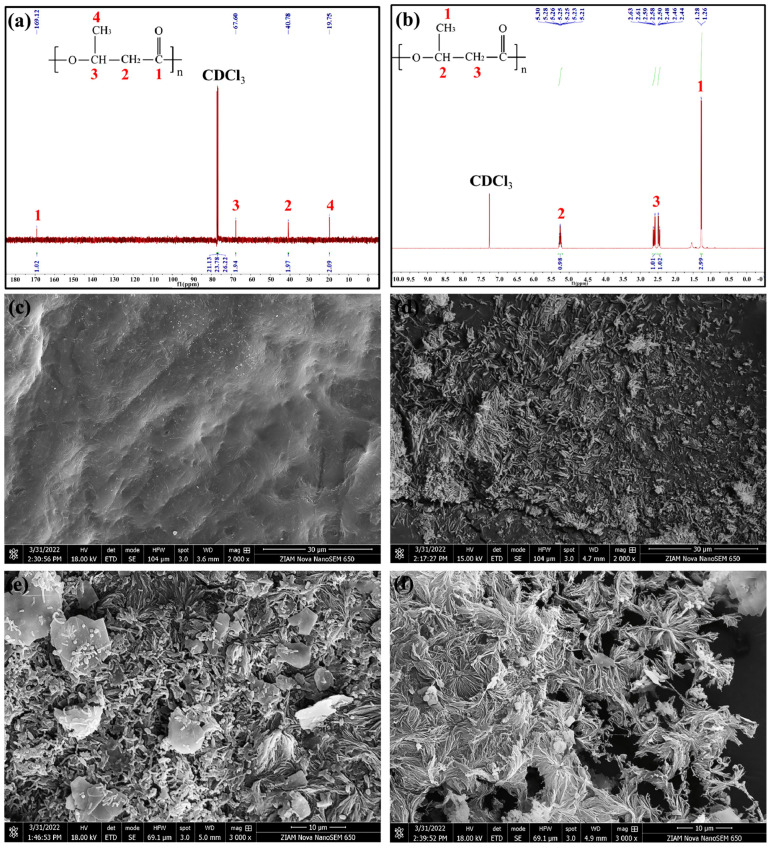
The characterization of extracted PHA from *S. thermodepolymerans* and commercial PHA. (**a**) ^13^C NMR and (**b**) ^1^H NMR of the extracted and commercial PHA. SEM images of the degradation of PHA by *S. thermodepolymerans* at 50 °C. (**c**) Initial PHA sheet, (**d**) *S. thermodepolymerans* immobilized on a solid surface after 2 days, (**e**) PHA sheet covered with *S. thermodepolymerans* after one week, (**f**) Small fragmented pieces of PHA sheets after two weeks with *S. thermodepolymerans* [91].

There are many other studies conducted on microorganisms. For example, Raunhan et al. utilized *Thauera mechernichensis* TL1 to convert food waste anaerobic digestate to PHA. The optimal molar C/N ratio for PHA production was first determined to be 20, and under this condition, the production of PHA was investigated when acetate, propionate, and food waste anaerobic digestate were used as carbon sources. Under these conditions, the production of PHA was studied when acetate, propionate and food waste anaerobic digestate were used as carbon sources. Substrate consumption, pH change, cell dry weight and PHA production were analyzed, and the results showed that the production of PHA was higher when acetate and propionate were used as carbon sources, while anaerobic digestion of food waste could also be used as a carbon source, although the production was lower. The structural and thermal properties of PHA were characterized by nuclear magnetic resonance spectroscopy (NMR) and differential scanning calorimetry (DSC), and it was found that polyhydroxy butyric acid (PHB) was produced when acetate was used as the carbon source and poly (3-hydroxybutyric acid-co-3-hydroxyvaleric acid) (PHBV) was produced when propionate and anaerobic digestion of food wastes were used as the carbon sources. In addition, the presence of the PHA biosynthesis pathway and related genes was confirmed after genome-wide analysis of *T. mechernichensis* TL1 [90]. In the case of the thermophilic bacterium *Schlegelella thermodepolymerans*, xylose was used for the production of PHA, and the effects of four variables, namely, pH, temperature, xylose concentration and C/N ratio, on the production of PHA were investigated. It was determined that the optimal conditions were 50 °C, an initial pH of 7.0, a concentration of xylose of 20 g/L, and a C/N ratio of 100. The PHA content in the dry weight of cells could reach 80 wt.% after 48 h. The results were summarized in the following table. After 48 h under these conditions, the PHA content in the dry weight of the cells could reach 80 wt.%. The product was identified as PHB by ^13^C NMR and ^1^H NMR spectroscopy, as shown in Figure 5a,b. Its thermal stability was comparable to that of commercial PHA. After monitoring the substrate consumption, pH changes and cell growth, it was found that the PHA content was highest at nitrogen concentration limitation and decreased after 48 h due to intracellular degradation. The metabolic pathways from xylose to PHA were identified using proteomic analysis, including the classical PHACAB pathway, the de novo fatty acid biosynthesis pathway and the fatty acid β-oxidation pathway. In addition, the bacterium was found to have an efficient degradation of extracellular PHA at 50 °C, as shown in Figure 5c–f. It was hypothesized that *S. thermodepolymerans* has an extracellular PHA depolymerizing enzyme encoded by PHAZ, which may be the main driver of the rapid degradation of PHA under thermophilic conditions [91].

In addition, the high production cost, unstable material properties and quality issues of PHAs limit their industrialization. Sabapathy et al. utilized mixed microbial cultures (MMCs) for the production of PHAs, and found that MMCs can utilize low-cost raw materials such as waste streams, and that factors such as cultivation conditions, substrate characteristics, feeding method, organic loading rate, and sludge residence time. Factors such as culture conditions, substrate characteristics, feeding method, organic loading rate, sludge residence time and microbial community structure significantly affect the production, and costs can be reduced by optimizing these factors [92]. In contrast, Tan et al. focused on next-generation industrial biotechnology (NGIB) based on extremophilic microorganisms, where halophilic *Halomonas* can produce PHAs in open, non-sterile, continuous fermentation, and molecular engineering modifications can improve yield and quality and co-produce high-value-added products, although its volumetric productivity is currently lower than that of conventional industrial biotechnology (CIB). Several companies are currently engaged in the industrial production of PHAs, partly utilizing CIB technology and a few with *Halomonas*-based NGIB technology. Each company is actively exploring low-cost substrates [93]. Some of the companies that commercialized PHAs are summarized in Table 1. In a comprehensive comparison, the microbial fermentation method has significant advantages in large-scale production, but the current production cost of PHA is still high, and further exploration of cheap raw materials and optimization of microbial strains and reaction systems are needed in the future.

Some progress has also been made in the recovery technology of PHA. Liu et al. proposed a sequential recovery strategy, i.e., decomposing the extracellular polymers using Na_2_CO_3_ first, and then digesting the non-PHA cellular material (NPCM) with rhamnolipids (RL). The study focused on sludge characteristics, recovery protocols, analytical methods, and optimization of reaction conditions through one-way experiments and response surface methodology, which resulted in a PHA recovery of 61.29% and a purity of 97.55 ± 0.92% at 0.9 RL/g TSS, 5 h, and 99 °C, along with an alginate recovery of 11.82 ± 0.62% as a by-product. Characterization of the recovered polymers showed good physicochemical, thermal, and mechanical properties with a more uniform molecular weight distribution. Moreover, the production cost of this strategy was only 14.61 CNY/kg, which was 58.6% lower than that of the CHCl3 extraction method. The recovered ALE by-products had an economic value. The carbon footprint analysis showed that this method performed better in terms of global warming potential and emissions. The sequential recovery strategy also provides a viable solution for downstream recovery and overall economics of the MMC PHA process [94].

Overall, significant progress has been made in the application of PHA in food packaging, with innovations in production technology and resource recovery further reducing costs, improving environmental sustainability, and demonstrating significant potential for commercialization.

#### 2.1.3. Chitosan

**Figure 6:**
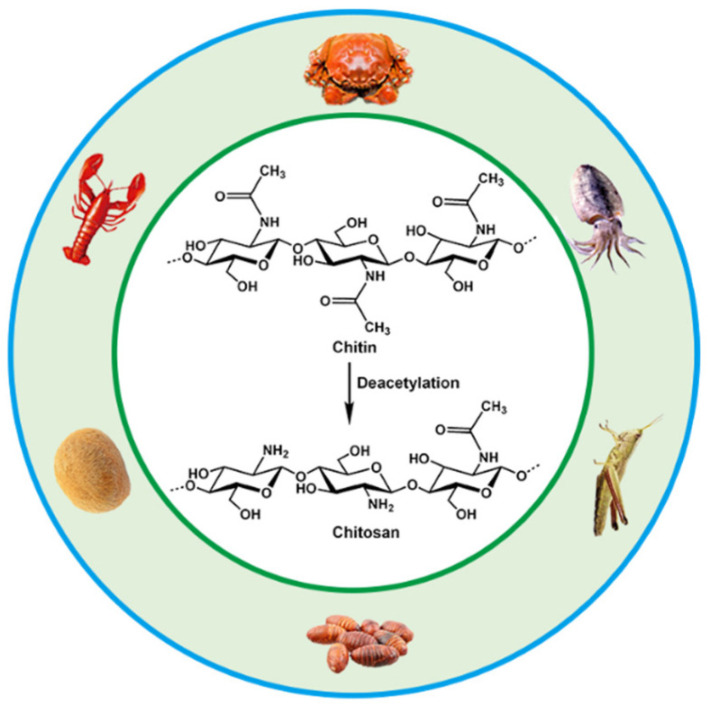
Sources and chemical structures of chitin and chitosan [96].

The preparation of chitosan as a natural polysaccharide compound begins with the deacetylation reaction of chitin. The degree of deacetylation significantly affects the application performance of polysaccharides in fields such as biomedicine and materials science by regulating their chemical composition, crystalline structure, thermal stability, and surface properties. For chitosan, a higher DD confers good hydrophilicity and antibacterial properties, making it more advantageous in practical applications. As the second largest renewable resource in nature, chitin is widely found in the shells of crustaceans and the exoskeletons of insects. We can also obtain it from insects such as bees, as illustrated in Figure 6 [95,96]. It can dissolve in diluted acids and form positively charged cationic groups, which interact with the anionic components of microbial cell walls and exhibit excellent antimicrobial, adsorptive, permeable, and hygroscopic properties [21,97,98]. Chitosan has a good affinity for human cells and does not cause rejection, and therefore is widely used in the fields of pharmaceuticals, food, and environmental protection. It is particularly used in the field of food packaging, where it is considered an ideal material to replace traditional petroleum-based plastics due to its degradability and biocompatibility. Chitosan has been used in the manufacture of food packaging for many years, which is regarded as an ideal material to replace traditional petroleum-based plastics [99,100].

In the field of food packaging, the development of novel antimicrobial packaging materials is crucial for ensuring food safety and extending the shelf life of food. Fu et al. prepared CS/pectin multilayer active food packaging films loaded with natamycin (NATA) and epigallocatechin gallate (EGCG) by layer-by-layer electrostatic self-assembly using chitosan (CS) and pectin as the film-forming matrix. It was found that the physicochemical properties of the packaging film were closely related to the addition of NATA and EGCG, and the CN/PE 15% film exhibited good UV shielding, and mechanical, gas barrier and antioxidant properties, which could effectively slow microbial growth and the quality deterioration of strawberries, having great potential for strawberry preservation [101]. In contrast, Lin et al. successfully loaded chrysanthemum essential oil (CHEO) into chitosan nanofibers (CS/NF) by electrostatic spinning to prepare CHEO/CS/NF nanofibers CHEO has strong antibacterial activity against *Listeria monocytogenes* (*L. monocytogenes*), which can disrupt the bacterial cell membranes, inhibit respiratory metabolism and is slowly released from the nanofibers to achieve long-lasting antimicrobial activity. It can be released slowly from the nanofibers to realize long-lasting antibacterial activity. The application of nanofibers in beef packaging can effectively inhibit the growth of *L. monocytogenes* in beef, delay lipid oxidation, and maintain the quality of beef, which is promising in the field of food packaging. These two studies provide new ideas and methods for the development of food packaging from different perspectives and promote the progress of food packaging technology [102].

Modifications of chitosan (e.g., alkylation, quaternization and phosphorylation) further enhance its antimicrobial, antioxidant and film-forming properties, and the derivatives are widely used in the preservation of fruits, vegetables, meat, and dairy products [103,104,105,106]. Cui et al. prepared chitosan nanoparticles (CSNPs) loaded with pomegranate peel extract (PE) by ionic gelation and added them to zein to make a composite membrane and used cold nitrogen plasma for surface modification of the composite membrane. The highest total phenol content and antioxidant activity were found in the PE extracted with methanol assisted by ultrasound. The CSNPs/PE nanocomplexes were spherical in shape, and the loading of PE led to an increase in the size of the nanoparticles and a decrease in the zeta potential. In the composite film, the incorporation of PE enhanced the tensile strength (TS) and elongation at break (EAB). The plasma treatment further improved these properties and thermal stability, while slowing down the release rate of PE and enhancing the inhibition of *Listeria monocytogenes* [107]. Shi et al. utilized mung bean protein microfibrils and chitosan (organic nanofillers and polysaccharides) in a composite through in situ fiberization and thermal–acid treatment, with the resulting bioplastic film exhibiting excellent CO_2_/O_2_ selectivity (coefficient of approximately 130) and mechanical properties (tensile elongation at break of approximately 230%). The heterogeneous, multi-dispersed nanostructure forms a dense microstructure, which not only optimizes gas barrier properties but also endows the material with the ability to carry 5 kg of fruit, offering a new solution for food preservation packaging [108].

**Figure 7:**
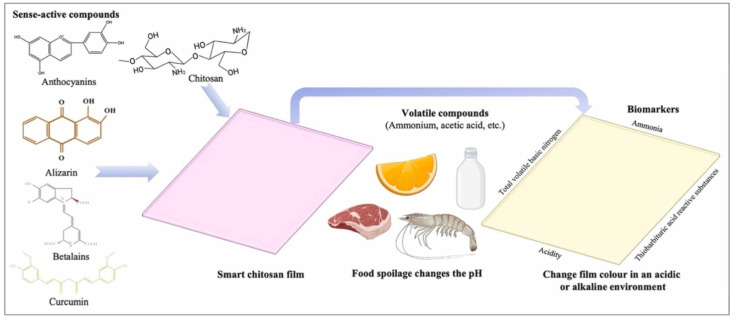
Different active compounds for the preparation of smart chitosan films with different freshness markers to assess the freshness of packaged products [111].

With the development of smart technology, chitosan smart films are also gradually being applied to food packaging, which can monitor the freshness of food and respond to changes in biomarkers, such as ammonia and pH, through sensory active compounds [109,110,111,112,113]. As shown in Figure 7, different active compounds were used to prepare smart chitosan films and to assess the freshness of packaged products by different freshness markers. Flórez et al. stated that chitosan can be used to prepare active and smart films, and the addition of active compounds can change the mechanical, barrier and functional properties of the films, e.g., phenolic compounds can enhance the mechanical and barrier properties, and essential oils, phenolic compounds, and fruit extracts can enhance antimicrobial and antioxidant capabilities. However, further research is needed to investigate their combination with other materials and the types of food products for which they are suitable [114]. Bhowmik et al. explored in detail the mechanism of smart chitosan film for monitoring food freshness biomarkers, describing its preparation method, the role of natural sensing active compounds, biodegradation pattern, cytotoxicity and safety issues. At the same time, they pointed out that there are challenges in the film’s ability to detect early food quality changes, color change sensitivity and application scope, which need to be studied in depth to achieve wider It is also pointed out that there are challenges in the detection of early food quality changes, the sensitivity of color change and the scope of application of this film, and in-depth research is needed to achieve a wider range of commercial applications [111]. Subramani et al. found that the food packaging films formed by compositing chitosan with biovanillin and kaolin showed excellent antioxidant, antibacterial and antifungal properties, along with good water vapor barrier and high degradability, demonstrating great potential as green food packaging materials [115]. The application of chitosan and its modified materials in the field of food packaging is promising and provides important support for the development of environmentally friendly, smart, and efficient packaging solutions. Table 2 lists some of the applications of chitosan-based films in different packaging applications by adding different compositions.

#### 2.1.4. Cellulose

Cellulose-based bioplastics are biodegradable plastics using cellulose, one of the most abundant natural macromolecules in nature, which combines renewability and excellent biodegradability [122,123,124,125]. These materials not only inherit the properties of traditional plastics, such as lightweight, waterproof, and durability, but also have significant advantages in terms of environmental friendliness and sustainability [39,126,127,128,129]. They are widely used in packaging, textiles, consumer products, and agriculture [130,131,132,133]. In recent years, cellulose-based bioplastics have been widely used in food packaging, and many related studies have focused on improving the performance of bioplastics, such as by adding lignin as a reinforcing filler to improve the mechanical strength or UV stability of cellulose-based materials, thereby developing more efficient bioplastics.

In the field of food packaging, it is crucial to develop packaging materials that are both environmentally friendly and functional. Ormanli et al. used electrospray technology to prepare a new cellulose-based active packaging material by loading paper with fulvic acid (FA) and silk gum (S), which have antioxidant and antimicrobial properties, and investigated the effect of the material on the quality of pears, which was found to delay the ripening and decay of the pears and extend the shelf life of the product. It was found that the material could delay the ripening and decay of pears and extend the shelf life of the products [134]. Shen et al. investigated the effects of various additives on film properties, such as in blended films, additives, such as tea polyphenols, and geranylgeranyl acetic acid, which were found to improve a variety of film properties. In composite films, nanoparticles, such as Zn O and TiO_2,_ can enhance the mechanical, antimicrobial, and antioxidant properties of films. However, due to less research on the cytotoxicity aspect of active films, the problems of nanofiller leakage and extract evaporation are yet to be solved. This can be improved by adding stabilizers or covalently grafted essential oils [135].

**Figure 8:**
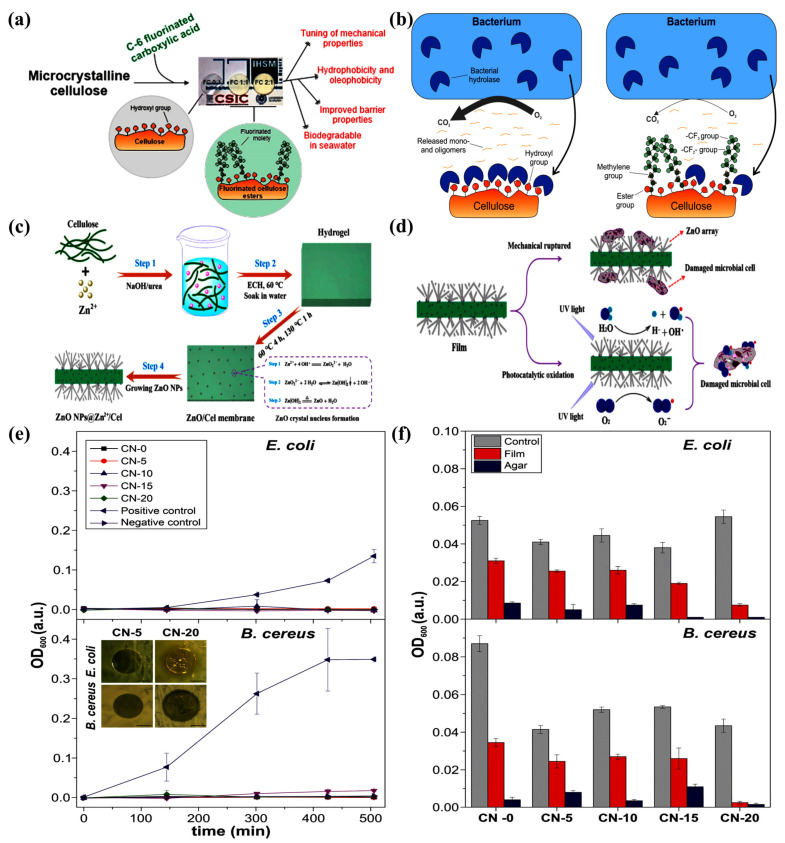
(**a**) Process for the preparation of cellulose-based bioplastics with excellent properties via C6-fluorinated carboxylic acid. (**b**) Scheme representing the biodegradation in seawater of pure (FC 0:1) cellulose and C6-fluorinated cellulose esters (FC 1:1 and 2:1) by a generic bacterium [131]. (**c**) Preparation of Zn O/cellulose composite films. (**d**) Mechanism of the antimicrobial activity of the films. Reprinted from [135] with permission from American Chemical Society. (**e**) Infiltration assay of *E. coli* and *B. cereus* on CN bioplastics as a function of time. Inset: photographs of CN-5 and CN-20 showing the bacterial inhibition. Scale bar: 0.5 cm. (**f**) Bacterial retentivity tests of CN samples for *E. coli* and *B. cereus* [137].

In terms of the preparation process, Benitez et al. prepared transparent cellulose–glycerol (CG) bioplastic films by dissolving cellulose with a high percentage of glycerol (up to 50 wt.%) in a mixed solvent of trifluoroacetic acid/trifluoroacetic anhydride and using a drop-casting method. It was found that glycerol formed intermolecular interactions with cellulose through a hydrogen-bonding network, resulting in an amorphous structure of the film, which significantly enhanced the flexibility (elongation at break increased from 4% to 49%) and toughness of the material while maintaining about 90% of the visible light transmittance. Thermal stability tests show that the effective combination of glycerin and cellulose increases the thermal degradation temperature from 150 °C to 176.8 °C. In terms of barrier properties, the film exhibits high water vapor transmission (~10,701 g/m^2^/day) and low oxygen transmission (~494 mL/m^2^/day), which is consistent with the needs of packaging for bakery products, and the overall migration (up to 6.4 mg/dm^2^) is lower than the EU standard. dm^2^ is below the EU standard limit. Biodegradation experiments show that glycerol significantly accelerates the degradation rate of the material in seawater, with a weight loss of 77.5% in 30 days and visible signs of microbial erosion on the surface. Application verification showed that the CG film could effectively delay the hardening of the sponge cake by up to 60%, and the oil resistance improved significantly with increasing glycerol content (Kit test value up to 7.5). The study also demonstrated that glycerol as a plasticizer can synergistically optimize the mechanical properties, barrier properties and biodegradability of cellulose-based materials, providing a viable alternative to traditional plastics, especially for food packaging with high respiratory demands [136]. Furthermore, Guzman-Puyol et al. prepared novel active food packaging films by dissolving casting and evaporating the solvent from microcrystalline cellulose with naringin in a mixture of trifluoroacetic acid and trifluoroacetic anhydride (2:1). The effects of different naringin contents on the optical, thermal, mechanical, antioxidant, antimicrobial, barrier properties and biodegradability of the films were systematically investigated and compared with other common food packaging polymers. The final results revealed that the bioplastic had excellent transparency and strong blocking ability against UV-A and UV-B, especially the latter, and the addition of naringin could modulate the mechanical properties of the cellulosic polymer matrix, enhance the antioxidant and antimicrobial properties of the material, and its barrier properties were similar to those of typical petroleum-based plastics and cellulosic derivatives used for food packaging; and its biodegradability in seawater was good. Combining all the properties, naringin-containing cellulosic bioplastics, especially CN-20, are expected to be a biodegradable alternative material for active food packaging. Figure 8 shows the preparation of some composite membranes and the mechanism and results of the antibacterial reaction [137].

Cellulose-based bioplastics can often be chemically modified or functionalized to improve their application properties, as in the case of Baniasadi et al. who, through a multi-component synergistic effect to achieve multifunctional integration of materials, constructed a ternary composite system of carboxymethylcellulose (CMC)/pomegranate peels (PE)/clay nanosheets, and successfully prepared multifunctional packaging materials with antimicrobial, antioxidant, and high-barrier properties. A multifunctional packaging material with antimicrobial, antioxidant and high barrier properties was successfully prepared. The experimental data showed that the tensile strength of the composite material was 300% higher than that of the pure CMC film with the addition of 3 wt.% of clay, and the water vapor transmission rate (WVP) and oxygen transmission rate (OTR) were reduced by 60% and 30%, respectively. Meanwhile, the introduction of PE gives the material broad-spectrum antimicrobial activity, with 91% inhibition of *Staphylococcus aureus* and *Listeria monocytogenes*, and its antioxidant properties remain stable for up to 24 h. Life Cycle Assessment (LCA) shows that the material has a low Global Warming Potential (GWP) of 4.4–4.8 kg CO_2_ eq./kg in a renewable energy scenario, with a 59% improvement in environmental benefits based on functional performance normalization [138]. Guzman-Puyol et al. focused on the hydrophobic and oil-resistant modification of C6-fluorinated carboxylic acid (TFNA) esterified cellulose through molecular design to balance the needs of material properties and biodegradation. The FC films catalytically synthesized by trifluoroacetic acid (TFA) exhibited excellent interfacial properties, including water contact angle up to 122°, oil repellency up to Kit Test 12, and oxygen permeability (55 mL/m^2^/day) and water vapor barrier (2331 g/m^2^/day) comparable to those of commercial petroleum-based materials polylactic acid (PLA) and polyvinyl chloride (PVC). It is noteworthy that despite the introduction of fluorinated chain segments to slow down the biodegradation kinetics, the FC film still exhibits significantly better biodegradation in the marine environment than conventional bio-based polymers (e.g., PLA), with a 30-day biological oxygen demand (BOD) of 11 mg O_2_/L and a weight loss of 34.9% [131].

Research and development of cellulose-based bioplastics for food packaging have focused on optimizing properties and expanding functionality, such as through the use of nanofibers (CNF), nanocrystals (CNC), and other modifications to enhance mechanical, barrier, and antimicrobial properties [130,139,140,141,142]. These modified cellulosic films are ideal solutions for sustainable food packaging, as they combine excellent tensile strength, low water vapor and oxygen permeability, and water- and oil-resistant properties. In the future, further optimization of their performance and cost will help drive the scaling up of cellulose-based bioplastics, providing new impetus to the environmentally friendly packaging industry.

### 2.2. Biobased Non-Biodegradable Bioplastics

#### 2.2.1. Bio-PE

The use of bio-based polyethylene (Bio-PE) in food packaging is gradually increasing due to its environmental friendliness and properties similar to those of traditional petroleum-based polyethylene [143,144,145,146,147]. Its raw materials can be derived from renewable resources such as sugarcane, which is recyclable and corrosion-resistant, and is suitable for use in food packaging containers and processing equipment, such as conveyor belts and loading containers; however, its promotion is still limited by cost and certification standards [148]. Polyethylene (PE), as a commonly used food packaging material, has the advantages of low cost and good processing performance, but it has problems such as poor oxygen barrier, the lack of an ultraviolet ray barrier, no antibacterial activity and unsustainable raw materials [149,150].

**Figure 9:**
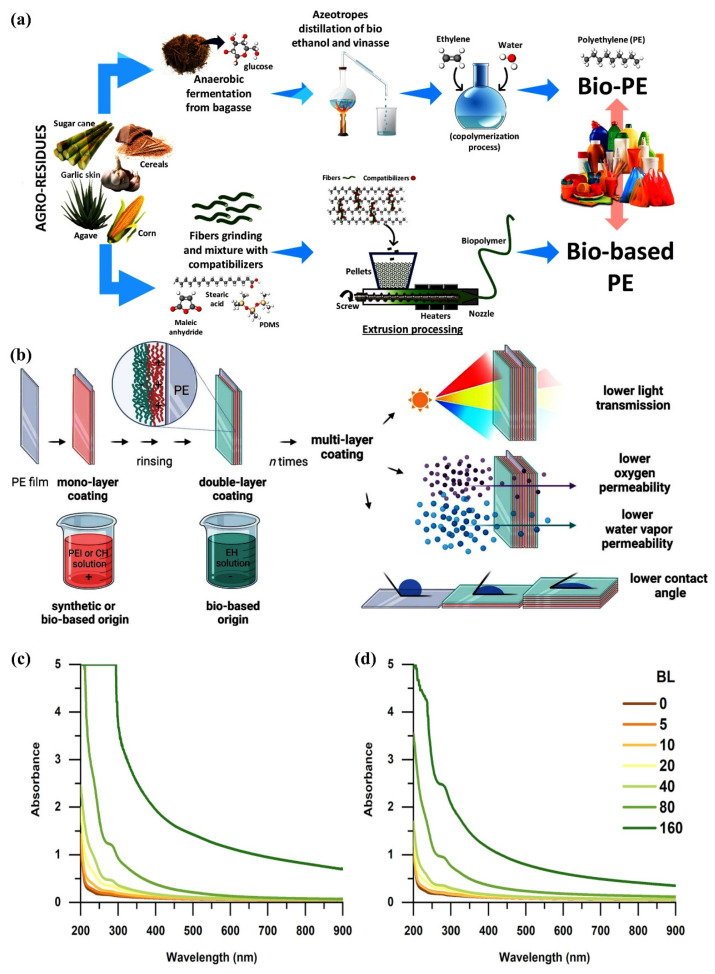
(**a**) Approaches of bio-PE and bio-based-PE from agro-industrial residues to the production of bioplastics in different disposable applications [143]. (**b**)The layer-by-layer assembly of a sustainably produced lignin-based polymer (EH) with polyethyleneimine (PEI) or chitosan (CH) was used to fabricate (partially or fully) bio-based coatings. Absorbance of PE films coated with a number of bilayers (BL) of PEI/EH (**c**) and CH/EH (**d**), ranging between 0 and 160. The absorbance of PEI/EH coated films with BL = 160 was out of range (higher than 5) in some of the UV region (200–294 nm) [151].

To obtain better materials for application, much research has been conducted on optimizing polyethylene films through different techniques aimed at enhancing the material properties. For example, Abbadessa et al. prepared partially or fully bio-based coatings that combine sustainably produced lignin-based polymers (EH) with polyethyleneimine (PEI) or chitosan (CH) using layer-by-layer assembly techniques, aiming to improve the barrier properties of PE films. It was shown that the light barrier and wettability of the coatings could be adjusted by varying the number of bilayers and that selected samples showed a significant improvement in oxygen and water vapor barrier properties, with the PEI/EH coatings performing better [151]. Lu et al. investigated the effect of silver ion injection on the antimicrobial properties of polyethylene (PE) food packaging films. It was found that low-dose silver ion injection can enhance the hydrophilicity of the film surface, inhibiting bacterial adherence and growth; the silver ions were found to be stable and do not color the film when the dose is less than 1 × 10^14^ cm^−2^, demonstrating the potential for application in food packaging [152]. Carullo et al. conducted an in-depth characterization of PE-based monolayer materials and compared their performance with traditional multilayer structures, and found that the monolayer materials have excellent performance in oxygen and water vapor barrier performance, and their coatings also have good UV-C shielding ability, tensile/puncture resistance and friction performance. At the same time, they have an advantage in the evaluation of environmental performance, which is expected to replace the traditional multilayer packaging materials. These studies provide theoretical support and practical reference for the development of food packaging materials from different perspectives and promote the development of the food packaging industry toward more environmental protection and high performance [153]. Figure 9 depicts the production and preparation processes of PE, along with its strengthening process and the ultimate strengthening outcomes.

#### 2.2.2. Bio-PET

**Figure 10:**
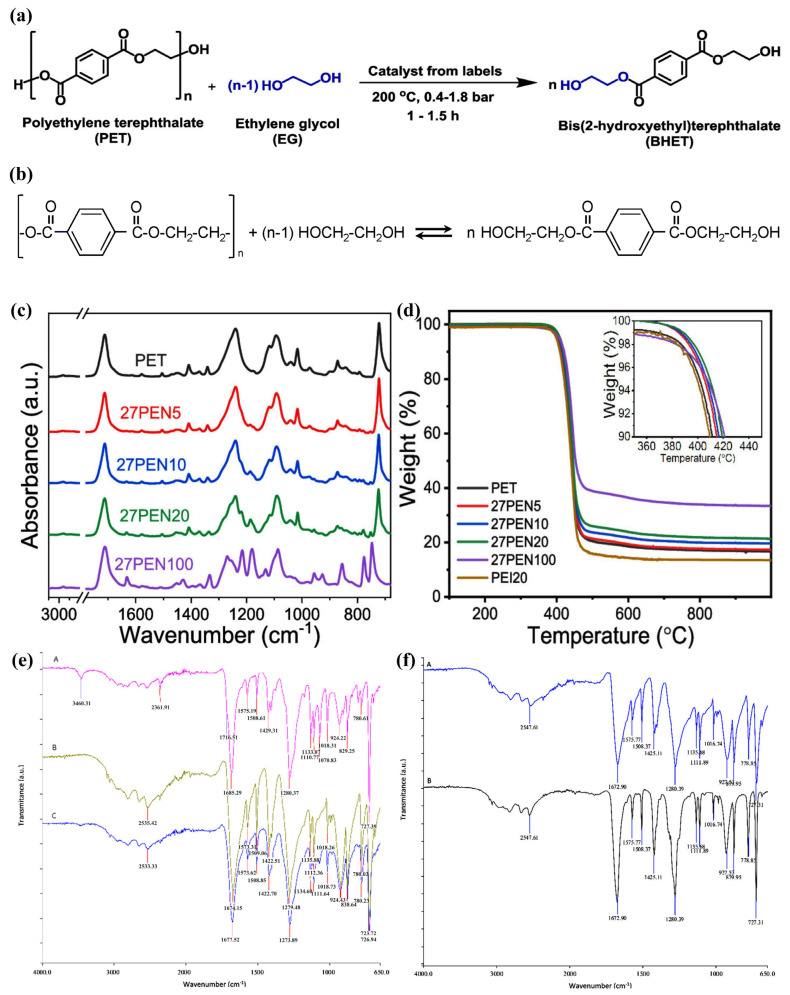
(**a**) PET glycolysis using a catalyst by the thermolysis of the labels from PET bottles [167]. (**b**) Depolymerization of PET to produce BHET using EG as a glycolysis agent [169]. (**c**) ATR-FTIR spectra of PET and naphtha late-based copolymers. (**d**) TGA thermograms with a heating rate of 10 °C min^−1^ [155]. (**e**) ATR–FTIR spectra of commercially available pure TPA (B–green spectrum), isolated solid fraction after neutral hydrolysis of PET foil (C—blue spectrum), and isolated solid fraction after neutral hydrolysis of PET foil coated with chitosan (A—pink spectrum). (**f**) ATR–FTIR spectra of commercially available pure TPA (A spectrum), isolated solid fraction after alkaline hydrolysis, and addition of acid of PET foil coated with chitosan (B spectrum) [168].

Biobased PET is a kind of polymer material made from biomass resources with resource-saving and low-carbon characteristics, which can replace traditional petrochemical plastics [154,155,156,157]. Prepared by copolymerizing the bio-based monomer terephthalic acid (PTA) with ethylene glycol (EG), it combines heat resistance, strength, modulus, and gas barrier properties, and can significantly reduce carbon emissions and environmental pollution. Although the cost is high, its environmental advantages offer it a broad development potential, and the waste can be converted into fuels and chemicals through pyrolysis or gasification, which supports the circular economy [55,158,159]. However, with the widespread use of PET materials in packaging and other areas, there has been much interest in whether their waste is incinerated, disposed of in landfills, or recycled twice, and it has been found that changes in waste management and consumption patterns can lead to environmental and social savings when quantified in comparison to conventional practices [160,161,162,163,164,165]. Figure 10a,b illustrates the process of PET depolymerization.

Rorrer et al. combined recycled PET with bio-based monomers to prepare high-performance, long-life composites by depolymerizing PET and reacting it with renewable monomers to synthesize unsaturated polyester (UPE) or diacritic acid polymers, and then making fiber-reinforced plastics (FRP). The experimental results show that the prepared r PET—FRPs outperform the standard petroleum-based materials, save 57% of supply chain energy, reduce greenhouse gas emissions by 40%, and are sold at a higher price. The experimental results show that r PET-FRPs can save 57% of supply chain energy, reduce 40% of greenhouse gas emissions, and can be sold at a higher price, which provides a new way to upgrade PET recycling and bio-economy development [166]. Lee et al. synthesized a series of PET copolymers containing the biobased dimethyl 2,7-naphthalene dicarboxylate (2,7-N) to improve the properties of PET. It was found that the addition of 2,7-N significantly improved the thermal stability, mechanical and barrier properties of the copolymers. The FTIR and TGA profiles of the prepared products are shown in Figure 10c,d. The glass transition temperature of the copolymers increased and the oxygen permeability decreased by 30% when the 2,7-N content was 20% [155].

In the field of PET waste recycling, several studies have explored innovative ways to achieve its effective utilization. Enayati et al. utilized the solid residue (containing calcium carbonate, calcium oxide and titanium dioxide) of PET water bottle labels after pyrolysis at high temperature as a catalyst for PET alcoholysis reaction, of which Cat-800 prepared at 800 °C had the best performance, with the conversion rate of PET up to 100% and the production rate of BHET up to 95.8% in 1.5 h, which provided a low-cost and environmentally friendly catalyst [167]. Volmajer et al. added chitosan as an active surface additive to PET materials to realize the antimicrobial function and chemical recycling of food packaging materials. Through neutral and alkaline hydrolysis experiments, it was found that chitosan coating can inhibit bacterial growth and completely depolymerize PET packaging materials into terephthalic acid and charcoal under alkaline hydrolysis conditions, which provides a new method for recycling complex PET wastes [168]. Figure 10e,f shows the FTIR spectra of the products derived from the alkaline and neutral hydrolysis of PET. A study examined the feasibility of alcoholysis for the chemical recovery of complex PET wastes from real household packaging wastes. The complex PET wastes were subjected to catalytic alcoholysis reaction by online identification and sorting of wastes through infrared spectroscopy (NIR). The results showed that the conversion rate of the alcoholysis reaction was significantly affected by time, temperature and EG/PET molar ratio, and that the alcoholysis products of high-color and multilayer PET wastes were similar to those of reference PET, which proves that alcoholysis is a promising strategy for the recovery of such wastes [169]. Listed in Table 3 are numerous studies dealing with the treatment of PET waste. All of them have created diversified channels for the sustainable management and resourceful utilization of PET waste.

**Table 3:** Some studies on the treatment of PET waste.

Add Ingredients	Function	Processing Technology	Degradation Rate	Reference
Co-CeO2	Catalyst	Photocatalytic reaction	91.61 ± 1.50%	[170]
TFA	Activator	Depolymerization reaction	96%	[171]
maleic acid/DSS	Test agent	Subcritical water conditions	100%	[172]
Glutathione S-Transferase	Catalyst	Catalytic reaction	98.9%	[173]
FAST-PETase hydrolase	Hydrolytic	Hydrolytic reaction	100%	[174]

### 2.3. Non-Biobased Biodegradable Bioplastics

#### 2.3.1. PBAT

**Figure 11:**
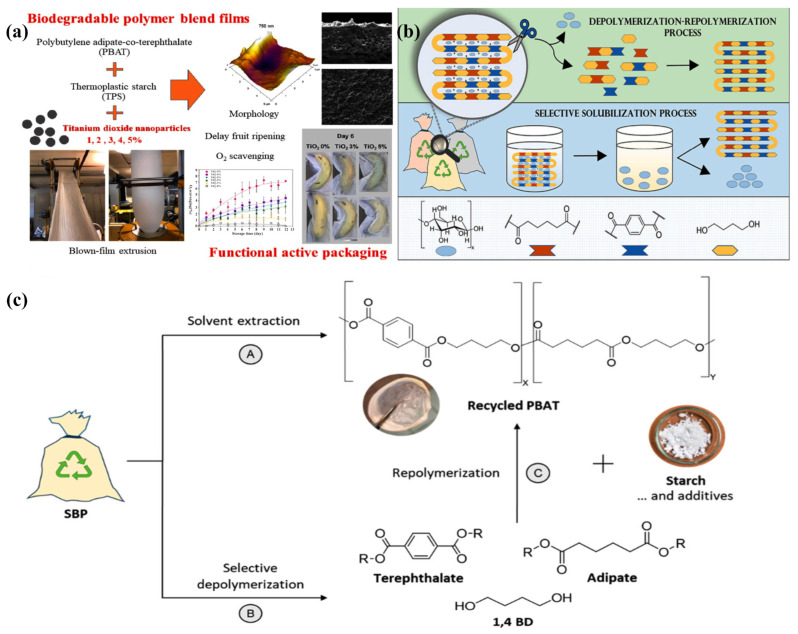
Preparation of PBAT-based various packaging films. (**a**) Schematic representation of PBAT/TPS-based functional composite blown film added with titanium dioxide nanoparticles [178]. (**b**) Schematic diagram of selective solubilization and cross-linking–decross-linking treatment of PBAT. (**c**) Investigated strategies for recycling SBPs (bottom): (A) selective solubilization; (B) depolymerization of PBAT to obtain DMT, DMA, and 1,4-BD; and (B) + (C) depolymerization–repolymerization process [185].

Poly (butylene adipate-co-terephthalate) (PBAT) is a thermoplastic biodegradable plastic copolymerized with butylene glycol adipate and butylene glycol terephthalate, which has good ductility, heat resistance and biodegradability, and can be processed by injection, extrusion, blow molding and other processes and is widely used in the fields of packaging, hygiene products and biomedicine [175,176,177,178,179]. Figure 11 presents a schematic diagram of a PBAT/TPS functional composite blown film, which is made by the addition of titanium dioxide nanoparticles. The global PBAT market size reached USD 1.46 billion in 2022 and is expected to continue growing in the future.

To optimize its performance as a bioplastic film for food packaging applications, PBAT is often modified by compounding to enhance its application potential. For example, Fernandes et al. conducted a study centered around a polyhydroxy butyrate (PHB)/polybutylene adipate-terephthalate (PBAT) bilayer membrane. At the beginning of the study, soil collection was carried out in accordance with strict standards, and the collected soil was subjected to comprehensive physical and chemical characterization. Subsequently, biodegradation experiments were carried out under specific temperature conditions using a specific reactor with a precise inoculum volume. The degradation of the bilayer film was scientifically evaluated by titrating the carbon dioxide production in different treatment groups. The membranes used in this experiment were prepared from specific materials and were used to ensure that the mass of organic carbon in each treatment group was equal in order to eliminate interfering factors. A variety of advanced analytical and microscopic methods, such as scanning electron microscopy (SEM), attenuated total reflection, Fourier transform infrared spectroscopy (ATR-FTIR), differential scanning calorimetry (DSC), and thermogravimetric analysis (TGA), were utilized in the study. At the same time, samples were collected to determine the microbial diversity, and at the end of the experiment, degraded microorganisms were isolated from the soil and subjected to a series of operations such as DNA extraction, polymerase chain reaction (PCR) amplification, sequencing, etc., to accurately identify microbial species, and also to determine the total DNA sequences of soil microbial communities to analyze the diversity in depth. Finally, the relevant data obtained were analyzed statistically, and appropriate tests were used to determine the significance of the differences in the data, and the results of the studies were combined to comprehensively assess the biodegradation characteristics of the double-layer film in soil and the microorganisms involved in the degradation process [180].

While Wang et al. focused on improving the performance of PBAT in food packaging applications. The lignin/zinc oxide composites were prepared by a hydrothermal method, in which different masses of lignin were reacted with zinc nitrate and triethylamine, respectively, and three products, L-Zn O-1, L-Zn O-2 and L-Zn O-3, were successfully obtained. Next, PBAT, epoxidized soybean oil and prepared L-Zn O were added to the torque rheometer for melt blending according to specific ratios, after which the films were made by the hot-pressing process. Subsequently, the prepared materials were systematically characterized and analyzed. Using X-ray diffraction (XRD) analysis, the presence of Zn O in L-Zn O-X was confirmed, and it was also shown that the additions did not change the crystal structure of PBAT, but rather improved its crystallinity. With the help of scanning electron microscope (SEM) observation, it was found that the particles were uniformly dispersed in the material with the addition of an appropriate amount of L-Zn O-1 and L-Zn O-2, while particle agglomeration occurred with the addition of L-Zn O-3. Therefore, P-L-Zn O-2 was selected for the subsequent in-depth study. The results of thermal property analysis showed that the thermal weight loss process of the composite films could be divided into two steps, and the addition of L-Zn O-X had a small effect on the thermal stability of the films. The differential scanning calorimetry (DSC) analysis showed that L-Zn O was able to promote the crystallization of PBAT. Finally, the performance tests were conducted. In the light aging experiments, P-L-Zn O-2 showed excellent anti-light aging performance, and its mechanism of action was to be able to trap free radicals and effectively block the degradation process. The barrier performance test found that the hydrophobicity and barrier performance of the film were both improved. Antimicrobial performance tests and strawberry packaging experiments show that the film has high antimicrobial activity and a good freshness preservation effect, with the potential to be applied as a food packaging material [181]. Similarly, Xiao et al. used acid-base adjusted hydrothermal method to prepare lignin-Zn O (L Zn) hybrid particles with different characteristics and used PBAT as the basic material to carry out the research, focusing on solving the problem of pollution of plastic packaging waste and improving the performance of food packaging materials, in order to solve the deficiencies of the antioxidant and antimicrobial properties of PBAT materials. Hybridized particles of L Zn were successfully prepared; their antimicrobial activity was enhanced with increasing Zn O content, e.g., the bacterial survival of L Zn-9 against *E. coli* and *S. aureus* was reduced to 9% and 49%, respectively. In terms of properties, the tensile modulus and yield strength of the PBAT-G-x l Zn films increased with increasing L Zn content, e.g., the tensile modulus of PBAT-G-3LZn was enhanced by 35.2% and the yield strength was enhanced by 28.6% compared with pure PBAT. In terms of antioxidant activity, although the free radical scavenging rate of PBAT-G-x L Zn films was lower than that of PBAT-x L Zn films, they still possessed a certain antioxidant capacity, and the free radical scavenging rate (RSA) of PBAT-G-3LZn was 11.6%. In terms of antimicrobial properties, the composite films had contact antimicrobial properties, and the bacterial adhesion rate of the PBAT-G-x L Zn films was significantly reduced. The hydrophobicity and water vapor barrier of the films were also improved by the incorporation of L Zn hybridized particles [182].

Moreover, Venkatesan et al. selected biodegradable PBAT and introduced N- and P-doped carbons (NPCs) to prepare composites. NPCs were synthesized by using urine as raw material, and then composite films were made with PBAT by the solution-casting method. Comprehensive analysis of the NPCs and the composite films using various characterization means showed that the NPCs had specific structures and morphologies, and their Fourier transform infrared spectroscopy (FTIR), X-ray diffraction pattern (XRD), scanning electron microscopy (SEM) and transmission electron microscopy (TEM) images fully confirmed the successful synthesis of NPCs and the uniform distribution of elements in them. In the tensile test, it was shown that the tensile strength and elongation at break of the composites were improved due to the plasticizing effect of NPCs; in the antimicrobial property evaluation, the PBAT/NPCs films exhibited significant antimicrobial activity against *E. coli* and *S. aureus*, while the PBAT films did not have such antimicrobial effect. The prepared composites exhibited excellent performance in terms of tensile strength, gas and water vapor barrier, and antimicrobial properties [183].

There are many common processing methods for PBAT bioplastics, such as blown film extrusion and flat sheet extrusion, which are effective in preparing films with stable properties. Wongphan et al. used the blown film extrusion method with thermoplastic potato starch (TPS) and polybutylene adipate/terephthalate (PBAT) as raw materials. When adding 1–5% sodium nitrite and sodium erythorbate to the composite film, it was found that both additives improved the processing efficiency of TPS through hydrolysis, enhanced the compatibility between TPS and PBAT, resulted in a smoother microstructure of the film, improved oxygen barrier properties, and reduced discoloration of packaged cooked meat stored at 4 °C for 9 days [176]. Castro et al. used PBAT/PLA to prepare films by the addition of 5 wt.% of turmeric or cinnamon powder by the platelet extrusion method. It was found that turmeric and cinnamon powders were thermally stable, and the addition of turmeric and cinnamon changed the film properties; for example, cinnamon reduced the UV light transmittance of the film, made the surface more hydrophobic, and the reprocessing improved the tensile strength and elongation at break of the film [184].

With the wide application of PBAT, people have also found some limitations in its recycling. The current common treatment is composting, which is not only a waste of resources but also not in line with today’s concept of “carbon-neutral” development of the circular economy. Parodi et al. proposed three strategies for the recovery of SBPs: first, a physical recovery method by selective dissolution of PBAT with ethyl acetate; second, a depolymerization–repolymerization chemical recovery method by catalytic selective alcoholysis of PBAT to oligomers, which were then polymerized to obtain PBAT; and third, a completely selective depolymerization of PBAT for the recovery of 1,4-butanediol (1,4-BD), dimethyl terephthalate (DMT) and dimethyl adipate (DMA). The processing related to it is illustrated in Figure 11b,c. By experimenting with each recycling strategy using SBP bags as raw materials, it was found that the three methods achieved 99%, 95%, and 93% recovery of components, respectively. The recycling strategy of combining chemical and mechanical recycling is conducive to reducing the waste of resources and environmental impacts, but we still need to further carry out a complete life cycle analysis (LCA) to clarify its actual impacts as well as the applicable boundaries. It is believed that with its degradability, functional diversity and recycling feasibility, PBAT will become an ideal choice for sustainable packaging and is expected to realize a wider application in the field of environmental protection and food packaging in the future [185].

#### 2.3.2. PCL

Bioplastic polycaprolactone (PCL) is a material with a low melting point, high biodegradability, and biocompatibility, which can be decomposed into harmless substances within a certain period and is widely used in the fields of drug carriers, degradable plastics and food packaging [186,187,188]. Its good intersolubility allows it to be combined with other polymer materials, and it can be used for modification or functional enhancement of bioplastics and so on. In addition, PCL also shows great application potential in the field of infrared modification. Through methods such as composite with photothermal agents, introduction of fluorescent groups, or cross-linking modification, the material can be endowed with specific infrared response characteristics to meet different application requirements. With the enhancement of environmental protection consciousness, the development of environmentally friendly food packaging materials has become a research hotspot. The composite of PCL and other materials can effectively improve its performance to meet the needs of food packaging involving environmental protection, antimicrobial properties, preservation of freshness and other aspects of the development of a new type of food packaging material to provide a theoretical basis and practical reference.

In related research, thermoplastic glutenin (TPG) and PCL have received attention as alternative bio-based materials. TPG is a material with film-forming ability, although it has water sensitivity and brittleness problems, while PCL is a hydrophobic, biodegradable polymer. The blending of the two is expected to improve the properties, and chromium octanoate was also used as a potential food-grade catalyst. By reactive extrusion and thermoforming techniques, the blended films of TPG and PCL with chromium octanoate as a catalyst were prepared. The TPG/PCL films were mechanically compatible despite the phase separation, and the addition of the catalyst facilitated the cross-linking reaction, which resulted in a more hydrophobic and crystalline material, and all the films were biodegradable and non-ecotoxic within 90 days, which is expected to be used as a shape-memory food packaging material [189]. Hu et al. prepared PCL/fucose nanofiber membrane loaded HP-β-CD/epigallocatechin (EGC) inclusion complexes using ultrasound-mediated and electrostatic spinning techniques. The material was successfully prepared with the characteristics of increased average diameter, enhanced water vapor permeability, reduced crystallinity and hydrophilic surface, and slow release of EGC, which showed excellent antimicrobial properties both in vitro and in vivo [190]. To address the problems of low melting point and poor mechanical properties of PCL, Gürler et al. blended waste photopolymer (WPP) with PCL and prepared PCL/WPP blended films with different WPP contents by the solution-casting method. This showed that with the increase in the WPP content, the films exhibited a decrease in thermal stability, an increase in the glass transition temperature, a decrease in transparency, an improvement in the permeability of water vapor, and shape memory properties, which can be used as potential food packaging materials [191]. These studies provide a theoretical and practical basis for the development of PCL-based food packaging materials from different perspectives and promote the development of environmentally friendly food packaging materials.

Foods such as fresh fruits are often more susceptible to contamination by *Escherichia coli* or *Staphylococcus aureus* during transportation and preservation, which results in high susceptibility to rotting and damage, causing economic waste. While cellulose nanocrystals (CNC) were prepared by acid hydrolysis, and modified cellulose nanocrystals (MCNC) were prepared through modification, and then PCL was compounded with MCNC, and bio-nanofilms obtained by solution casting method were found to be significantly strengthened in mechanical properties, ultraviolet blocking and antimicrobial effects after the addition of 3 wt.% of MCNC, and they exhibited Good antimicrobial properties were found, and the freshness could be maintained for 22 days in cherry preservation experiments [192]. Another innovation of PCL is the combination of PCL with polylactic acid (PLA) by incorporating oregano essential oil (OEO) and β-cyclodextrin (β-CD) inclusion through electrospinning technology to produce a reactive packaging material that excels in thermal stability and mechanical properties, especially 6 wt.% OEO@β-CD/PLA/PCL nanofibers, which have a modulus of elasticity of 21.1 MPa. In addition, these nanofibers had good hydrophobicity with a water contact angle of 116.59° and low water vapor permeability, which effectively retarded the water loss and quality degradation of blackberries during storage. The OEO@β-CD/PLA/PCL nanofibers exhibited significant antimicrobial activity against *Escherichia coli* and *Staphylococcus aureus*, as well as good antifungal activity against *Staphylococcus aureus* and Bread mold. In blackberry preservation experiments, OEO@β-CD/PLA/PCL nanofibers significantly reduced the decay rate of blackberries, maintained the appearance and quality of blackberries, and effectively delayed postharvest decay of blackberries [193]. These studies highlight the multifunctionality of PCL in food packaging, which, through compounding with other materials or process optimization, can balance environmental friendliness, functionality and practicality, and promote the development of sustainable and active food packaging materials.

#### 2.3.3. PBS

Bioplastic polybutylene succinate (PBS) is made by the polymerization of succinic acid and butylene glycol, which has complete biodegradability, excellent processing performance, mechanical strength, biocompatibility and heat resistance. The products produced from its degradation are non-toxic and harmless, which is of great significance to environmental protection. It is widely used in the fields of food packaging, agricultural mulch and medical materials [194,195,196,197,198,199].

In the food packaging field, PBS is often compounded with other materials to develop different functional films, for example, to develop materials with properties such as high biodegradability and antimicrobial properties. Zabidi et al. blended PBS with polylactic acid (PLA) and added nanofibrillar cellulose (NFC) and essential oil of thyme (EO) to form active bio-nanocomposite films. Different ratios of PLA/PBS films were prepared and after determining the optimum ratios, the addition of different levels of NFC (1.0–3.0%) and 9% of EO gave PLA/PBS/NFC/9T films were obtained by adding different levels of NFC (1.0–3.0%) and 9% EO. Through its characterization, it was found that EO had good compatibility with other components, and that excessive NFC would lead to agglomeration and decrease the tensile strength (TS) of the films, while 2% NFC could increase the tensile strength (TS) of PLA/PBS films by 12%. NFC can increase the film thickness, NFC can change the color and transparency of the film, and EO can increase the transparency. NFC and EO can improve the thermal stability of the film. The addition of NFC and EO can also accelerate the degradation of PLA/PBS film, and has antibacterial activity against *Staphylococcus aureus* and *Escherichia coli*, which can prolong the shelf-life of chicken breast meat [200].

PBS/LN, PBS/CN and PBS/LN/CN composite films prepared by Nuamduang et al. through the addition of lignin nanoparticles (LN) and trans-cinnamaldehyde (CN) by melt extrusion of blown film were found to increase the residual amount of CN in PBS/LN/CN composite films by adding LN as compared to pure PBS films. The two improved the thermal, mechanical and barrier properties of the films, and the PBS/LN/CN composite film showed excellent antimicrobial effect, which can effectively inhibit the growth of mango anthracnose, regulate the concentration of gases in the package, and slow down the loss of mango weight as well as address the problem of decreasing hardness. Although there is a problem of CN explanation in the processing process, the technology of lowering the extruding temperature and adopting the technology of embedding can help to reduce the loss, which can help to minimize the loss of fruit after picking [201]. Another study prepared LNPs from softwood kraft lignin, achieving high-value utilization of industrial by-products and reducing the environmental burden of lignin. Compounding lignin nanoparticles (LNPs) with cinnamaldehyde (CIN) also significantly improved the antifungal properties of the PBS-based material, and the synergistic effect of adding 1% LNP with 5% CIN was found to be significant against Penicillium spp. fungi, with an in vitro experimental inhibition rate of up to 75.93% and achieving complete inhibition of the growth of yeasts and molds in bread packaging. At the same time, the water vapor permeability (WVP) and oxygen permeability (OP) of PBS were also reduced, which was attributed to the hydrophobicity of LNPs and the zigzag path formed by the nanoscale dispersion [202].

To solve the defects of insufficient functionality of pure PBS films, the alkaline-treated halloysite nanotubes (a Hal) were introduced into the PBS matrix, and the PBS/a Hal nanocomposite films were prepared by solvent-casting and melt-blending methods, respectively. After various characterization tests on the a Hal powder and films, it was found that the alkaline treatment increased the diameter of the a Hal lumen and enhanced the adsorption capacity. In the films prepared by these two methods, a Hal agglomerated when the addition amount was more than 3 wt.%, and the films obtained by the melt-blending method had a higher degree of crystallinity, were more pliable, more durable, and more hydrophobic, with a lower water vapor permeability. The effect of the PBS/a Hal films on the inhibition of *Escherichia coli* was also very significant. The inhibition effect of PBS/a Hal films on *E. coli* was also significant. The addition of a Hal enhanced the ethylene scavenging ability of PBS, and the film with 5 wt.% a Hal had the best performance among the two preparation methods. In packaging tests for apples and tomatoes, films containing 5 wt.% a Hal were effective in extending the shelf life of the fruit and reducing weight loss [203]. While the PBS material was treated by plasma etching and SiO_2_ coating technology, the nanostructure of the prepared material destroys the cell membrane of bacteria through mechanical stress, and the inhibition rate of *Escherichia coli* and *Staphylococcus aureus* is >4log CFU/cm^2^. The SiO_2_ coating makes the oxygen transmittance rate (OTR) decrease by 30%, which is better than that of the pure PBS material, and at the same time, maintains good transparency. Low-temperature plasma treatment can avoid thermal damage to the material, which is suitable for large-scale production [204]. The introduction of SiO_2_ nanoparticles into the PBAT/PBS blend system by extrusion improves the physical and barrier properties of the material, and the addition of 1% SiO_2_ leads to a 26% reduction in WVTR, creating a “zigzag path” effect at the nanoparticle–polymer interface. The tensile strength (MD direction) increases by 10%, and the elongation at break (CD direction) decreases but remains flexible. With the uniform dispersion of nanoparticles, the light transmittance of the composite film remains above 60%, which is suitable for transparent packaging and meets the EU food contact standards [205]. To address the deficiencies of antioxidant as well as antimicrobial properties of PBS in food packaging applications, PBS composite films containing different concentrations of quercetin were prepared by the solvent-casting method. The introduction of quercetin significantly enhanced the antioxidant capacity of the films and showed some inhibitory effects against *Escherichia coli* and *Staphylococcus aureus*. Optical property tests showed that the composite films had a deeper color (lower L* value) and decreased transparency, but the UV-blocking ability was significantly enhanced (transmittance below 400 nm tended to be close to 0). In terms of mechanical properties, the high concentration of quercetin resulted in a decrease of 30% and 54% in tensile strength and elongation at break, respectively, but no significant change in the water vapor transmission rate. Thermal analysis showed that quercetin did not significantly affect the thermal decomposition temperature of PBS (about 399 °C), and its migration behavior was significantly affected by the polarity of the food matrix, with higher release in ethanol and alkaline solutions. By optimizing the formulation as well as the process, the PBS films were finally successfully endowed with excellent antioxidant and moderate antimicrobial properties [206].

These innovative solutions produce application-compliant films through strategies such as nanomaterial modification, surface engineering and natural active ingredient addition. They optimize the barrier, antimicrobial and freshness preservation functions of pure PBS materials, and promote the development of environmentally friendly active packaging materials by utilizing industrial wastes (e.g., lignin) or natural ingredients, providing an important technological pathway to reduce food waste, enhance safety and sustainable development.

## 3. Processing Technologies and Applications of Bioplastics

### 3.1. Thermoforming

Thermoforming is an important process in the field of modern plastics processing; its core value in the food packaging industry has been fully embodied, a large number of studies have confirmed that the technology, through the production of lunch boxes, food trays and other standardized products, can effectively meet the food packaging on health and safety and large-scale production needs. This broad applicability in industrial applications has laid a solid foundation [207,208,209]. The technology focuses on softening bioplastics by heating and applying pressure to shape them, and then cooling them to form the desired shape, thus improving the strength of the packaging material and ensuring stability in transportation and storage [210,211,212]. The advantages of this technology are that it is highly efficient, energy-saving, cost effective and widely used in the plastics industry [213,214,215].

Thermoforming technology has made significant progress in the field of biobased packaging through material innovation and process optimization. In biobased composites, the PLA/PBS blending system and the introduction of highly hygroscopic calcium carbonate (CaCO_3_) as a reinforcing filler were adopted, and through the crystalline modulation mechanism (the crystallinity was increased to 18.5%) and the optimization of thermal diffusivity, we have succeeded in realizing the process compatibility of traditional thermoforming equipment. At the same time, we have maintained thermal stability (T g = 61.8 °C) and low water absorption (0.4% in 72 h), breaking through the limitations of CaCO_3_ application in bio-based materials [216]. In terms of plant fiber-based molding materials, the dry thermoforming technology developed based on non-wood fibers, such as bagasse and bamboo fibers, shortens the production cycle to 5 s through fiber pre-compression and biopolymer lamination process, and combines with 3D printing molds to achieve complex shape molding. The product combines both high strength (tensile strength of 4.1–43.7 MPa) and thermal insulation (thermal conductivity 0.058–0.061 W/m-K), achieving environmental protection without chemical additives through lignin self-bonding effect and natural antimicrobial agents. Both technologies have achieved functional integration through material–process synergistic optimization. In the future, breakthroughs are needed in the improvement of stability of bio-based materials, the control of energy consumption in large-scale production and the construction of a circular economy system, while combining intelligent material design and digital manufacturing technology to accelerate the process of replacing traditional plastic packaging [211].

Thermoforming in biomass-based materials can also provide an important technological solution to plastic pollution and resource recycling through molecular design and process optimization. Jia et al. focused on molecular design, closed-loop recycling and synthesized light-responsive Xylan cinnamate (XC) through ionic liquid-mediated esterification reaction, with a glass transition temperature adjustable in the range of 65–150 °C and prepared XC by the hot-pressing process, which combines high mechanical strength (tensile strength of 25–50 MPa), water resistance (water contact angle of 79 ± 0.88°), and transparency (visible light transmittance of >150%). The closed-loop recyclable plastic with high mechanical strength (tensile strength of 25–50 MPa), water resistance (water contact angle of 79 ± 0.88°), transparency (visible light transmittance >94%), and UV-shielding properties was prepared by the hot-pressing process. The photodimerization reaction of XC enhances the strength of XC by 100% after UV irradiation and the ionic liquid can be recycled, which, in combination with its complete soil degradation in 40 days and the 90% survival rate of the seed packages, demonstrates the unique advantages of the biomass material in the field of food packaging [217]. On the other hand, Friedrich et al. focused on process–structure synergistic optimization by studying the thermo-compression mechanism of wood–plastic composites (WPC), revealing that the material structure can be significantly optimized by the 190–210 °C thermo-compression process: closing the pores on the surface increases the density to 1.08 g/cm^3^, the contact angle reaches 88.4°, and at the same time, it enhances the surface hardness (Brinell hardness (Brinell +25%), flexural strength (MOR +20%) and impact resistance, especially for materials with high fiber content (60–70%) [218].

### 3.2. Injection Molding

**Figure 12:**
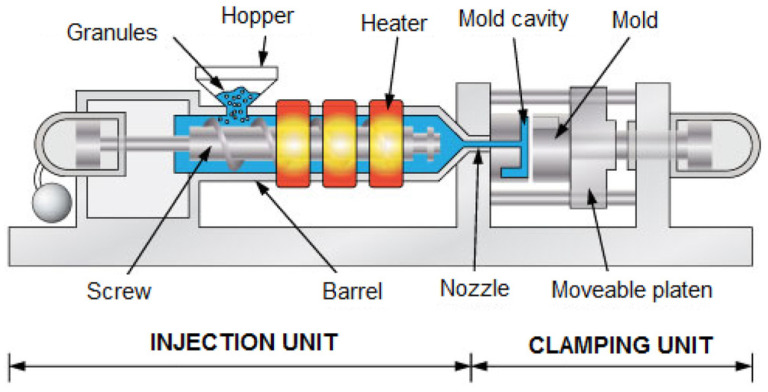
Simple schematic of injection molding machine [229].

As a widely used plastics processing technology in modern industry, the core of the injection molding process lies in the precise control of heating the temperature and injection pressure to inject molten plastic into the mold after cooling and curing to obtain a specific product shape [219,220,221,222,223]. Figure 12 presents common injection molding machines. Studies have shown that the precise regulation of process parameters not only directly affects the dimensional accuracy of the product but also has a significant positive correlation with the physical properties of the final product, such as mechanical strength and heat resistance [224,225,226,227]. This precision processing characteristic makes the traditional injection molding process important in industrial manufacturing, but its dependence on petroleum-based plastics also produces significant environmental problems. In this context, the injection molding process of bioplastics has emerged to provide an innovative solution to the bottleneck of the traditional process. Compared with petroleum-based plastics, bioplastics can be more environmentally friendly and reduce environmental impact by adopting the injection molding process of bioplastics from renewable resources at the raw material end [228]. By improving the injection molding parameters to match the characteristics of biomaterials, this technology has been successfully industrialized, especially in the food packaging field. This technological breakthrough not only continues the precision manufacturing advantages of the injection molding process but also builds a full life cycle environmental protection system from production to disposal through material innovation and promotes the transformation of the plastics processing industry toward sustainable development.

Injection molding, as an efficient processing means, has significant advantages in the field of bioplastics, which can reduce costs by using agricultural wastes (e.g., rice bran, cereal husk), and at the same time, regulate the material properties through the optimization of process parameters (e.g., temperature, pressure). It can provide theoretical support for exploring raw material selection, modification methods and the structure–property relationship for sustainable material development. In the preparation of rice bran-based bioplastics, it was found that the steam-cooked rice bran exhibited optimal mechanical properties (30% increase in viscoelastic modulus and 0.81 MPa tensile strength) due to its high protein content (19.42%) and more complete destruction of the starch structure. Its dense structure reduced water absorption to 171%, and it could be degraded within 30 days under a composting environment [230]. The composites prepared by blending grain hulls (FMH) with PLA/PBAT through injection molding partially improved the interfacial compatibility by the corn alcohol soluble protein coupling agent, which resulted in the thermal stability of the composites (Tmax up to 409.63 °C) and an increase in the biodegradation rate up to 13.11% in 180 days; however, the high filler content (>15%) resulted in the decrease in the impact strength [231].

In terms of performance optimization, plasticizers have a significant impact on bioplastic properties, which can be significantly enhanced through plasticizer system optimization, fiber reinforcement modification and process parameter regulation. Tábi et al. focused on the modification of PLA, pointing out that plasticizers (e.g., OLA) can enhance ductility, PBAT blending needs to improve the interface through GMA, and the combination of a nucleating agent (e.g., Zn PP) and a three-dimensional composite technology (PLLA/PDLA) can break the HDT bottleneck (up to 150 °C). Plasticization, impact modification, blending or preparation of biocomposites can improve the ductility and heat deflection temperature of PLA injection-molded products, thus expanding their engineering potential [232]. In cassava starch-based bioplastics, the use of a glycerol/water composite plasticizing system (e.g., 30% glycerol + water) increased the tensile modulus of the material up to 22 MPa (compared to 9 MPa for the pure glycerol system), reduced the water absorption up to 188%, and revealed its softening behavior (modulus decreasing by 3 orders of magnitude) at 80% RH through humidity-controlled dynamic mechanical analysis (DMA-RH) [233]. In contrast, in the soybean protein-based material, the addition of 5 wt.% lignocellulosic fibers resulted in a 160% increase in tensile strength to 2.8 MPa and a 115% increase in elongation at break to 0.54 mm/mm, while water absorption decreased from 340% to 185%. Optimization of process parameters resulted in an 18% increase in material crystallinity and a 32% enhancement in interfacial bond strength when the mold temperature was raised to 130 °C [234].

The injection molding process can effectively balance the mechanical properties, thermal stability, degradability of bioplastics through the pretreatment of raw materials (e.g., cooking, coupling agent), interfacial regulation and optimization of the crystallization behavior, which provides a technical path for the high-value utilization of agricultural waste and helps the development of environmental protection. With the continuous development in the future, people need to further solve the problems of interfacial compatibility and large-scale production.

### 3.3. Extrusion Molding

**Figure 13:**
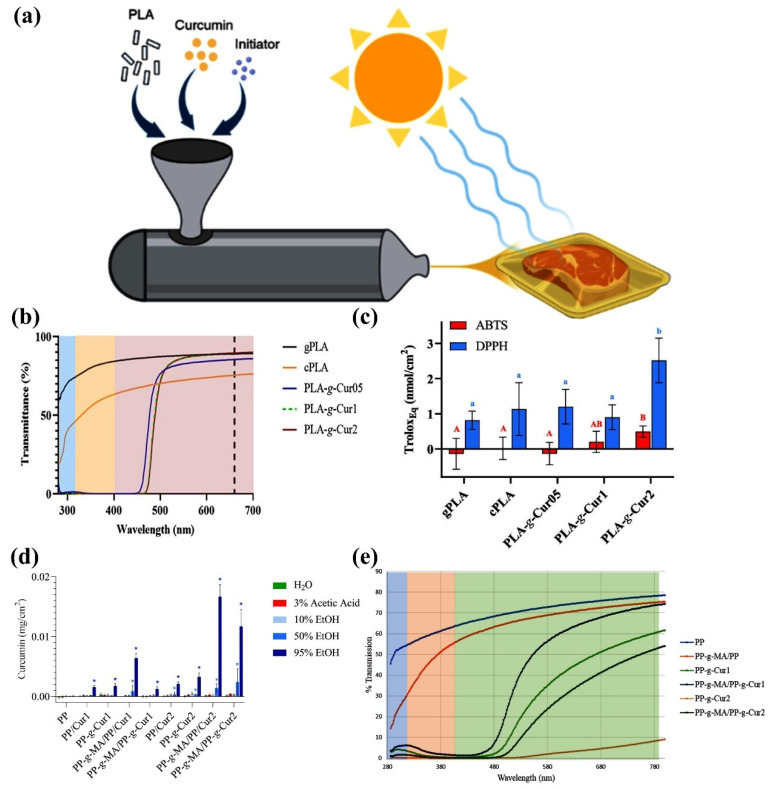
(**a**) PLA-g-Cur film for active and smart UV-blocking packaging with higher thermal stability via extrusion reaction. (**b**) UV–vis spectra of PLA-*g*-Cur and control films to demonstrate UV-blocking and optical transmittance properties of treated samples. (**c**) ABTS and DPPH radical scavenging assays to demonstrate the antioxidant capacity of PLA-*g*-Cur and control films [244]. (**d**) Migration study of treated and control films at 40 °C for 10 days in water, 3% acetic acid, 10% EtOH, 50% EtOH, and 95% EtOH. (**e**) UV–vis spectrophotometry spectra (280–800 nm) of treated and control films to demonstrate UV blocking and visible light transmission of treated films [243]. Color-coded letters indicate statistically significant differences between sample means for each assay (Tukey’s HSD, *p* ≤ 0.05). Statistically significant differences between the mean of each sample compared to the mean of PP for each simulant are signified by color-coded asterisks (Dunnett’s HSD, *p* ≤ 0.05).

The large-scale application of extrusion molding technology in the field of food packaging mainly relies on its unique process advantages and material suitability. By heating bioplastics to a molten state and then extruding them continuously through a mold, the technology can efficiently produce core products such as films, sheets and hollow packaging tubes, etc. [235,236,237,238]. Compared with the traditional intermittent production process, this continuous processing mode not only significantly improves the production efficiency, but also ensures that the material forms a homogeneous molecular arrangement structure during the process of phase change through the precise temperature control system and mold design [225,239,240,241,242]. Figure 13 illustrates the process of manufacturing PLA-g-Cur film with enhanced thermal stability for active and intelligent UV-blocking packaging through extrusion reaction. The food packaging materials produced by the extrusion molding process not only have good transparency, strength and barrier properties, but are also suitable for bio-based plastics processing, which promotes the popularity of biodegradable food packaging [243,244].

The latest research on extrusion molding technology in the field of bioplastics has significantly enhanced the performance and application potential of functional biomaterials through the optimization of process parameters and material innovation. Zhai et al. prepared LDPE—curcumin composite film by melt extrusion, which can inhibit curcumin exudation through hydrophobic structure, with a detection limit of 0.18 μM for ammonia (NH_3_) and achieve real-time visualization of meat spoilage (TVB-N value) real-time visualization monitoring, providing a safe and reliable intelligent solution for food packaging. The application of this natural pigment breaks through the toxicity limitations of traditional synthetic dyes while maintaining stability in high-humidity environments [245]. In the field of synthetic polymers, Shlush et al. achieved the first continuous production of ethyl cellulose (EC) by studying its hot-melt extrusion processing. By screening Myvacet^®^ as a highly efficient plasticizer (T g was reduced from 128.7 °C to 113.5 °C), the films produced had both high oxygen permeability and moderate water vapor permeability, which meets the demand for packaging of fresh agricultural products. It was also found that the molecular structure of the plasticizer (e.g., the long-chain structure of glyceryl monooleate) significantly affected the ductility of the material, with elongation at break reaching 62.7% [246]. For protein-based materials, the extrusion process was effective in modifying wheat gluten and soy protein by-products. In the wheat gluten system, extrusion significantly improved the compatibility and overall properties compared with compression molding, and alkaline conditions (pH 9) enhanced the tensile strength to 18.4 MPa by promoting the SH-SS cross-linking reaction, whereas the addition of xanthan gum or glyoxal could regulate the mechanical properties and water absorption of the materials. For the soy protein by-product, the mechanical strength and water absorption of the material could be precisely controlled by adjusting the extrusion temperature and screw speed. Optimizing the extrusion parameters (125 °C, 300 rpm) increased the tensile strength to 0.7 MPa while maintaining the high-water absorption of 70%, and the balance between S-S cross-linking promoted by the temperature increase and the degradation of the material became the key regulatory factor, with the high temperature enhancing the mechanical properties and the low temperature enhancing water absorption, making it suitable for industrial production. Enhanced mechanical properties at high temperatures and enhanced water absorption at low temperatures provide an important basis for industrialized production [247,248].

### 3.4. Coating Technology

**Figure 14:**
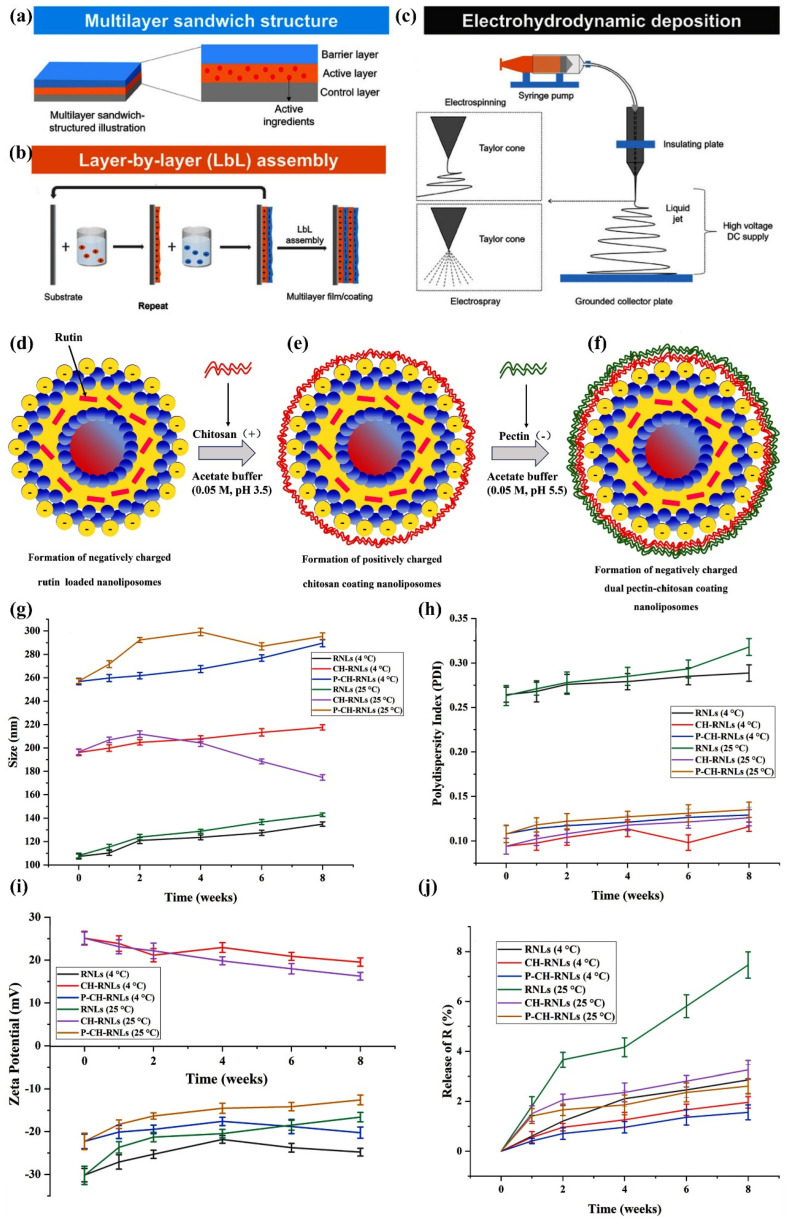
Multilayer edible coating methods. (**a**) Multilayer sandwich-structured edible coating with three-layer architecture comprising barrier, active, and control layers. (**b**) Layer-by-layer assembly using an electrodeposition approach based on interactions of natural bonds in the solution. (**c**) Electrohydrodynamic deposition is divided into electrospraying and electrospinning approaches [253]. Mechanism of nanoliposomes formation, (**d**) ruin loaded nanoliposomes, (**e**) chitosan-coated nanoliposomes, (**f**) pectin–chitosan dual-coated nanoliposomes. (**g**–**j**) Storage stability of nanoliposomes over 8 weeks at 4 °C and 25 °C [257].

In the field of food packaging, the application of coating technology can enhance the barrier, freshness, and antimicrobial properties of the material, and has become a key technical means to extend the shelf life of food [249,250,251,252]. In recent years, studies have shown that five main types of this technology system have been developed: aqueous coatings (e.g., starch, cellulose), hot-melt coatings (e.g., PLA with polylactic acid), natural wax coatings (e.g., beeswax), protein-based coatings (e.g., soybean protein), and plant-extract coatings (e.g., tea polyphenols) [253,254,255,256]. As presented in Figure 14a–c, this is a general technical sandwich structure of multi-layer edible coating technology, consisting of a three-layer structure comprising a barrier layer, an active layer, and a control layer. The application of these new coatings not only makes them excellent in blocking oxygen and water vapor, but also the biodegradable properties are more in line with the needs of sustainable industries.

Innovative research on coating technology in sustainable packaging has shown significant progress through multi-dimensional breakthroughs, focusing on innovative applications of coating technology in food preservation and environmentally friendly packaging. Hassane Hamadou et al. modulated the interfacial properties of nanoliposomes by electrostatic deposition of a pectin–chitosan double coating to provide a stable carrier for hydrophobic active ingredient delivery by reducing ruin encapsulation efficiency by 15.7% while improving oxidative stability by 40% and extending the storage cycle [257]. Figure 14d–f presents both the preparation process of nanoliposomes and the corresponding results. Esterified lignin/PBAT composite coating increases the water contact angle to 112° and reduces the oxygen transmission rate to 0.8 cm^3^/μm/(m^2^·day·kPa) by modifying dispersibility and breaking through the bottleneck of barrier performance of bio-based materials. The nano-emulsion-loaded plant essential oil technology enhances the antimicrobial activity by 60% through the interfacial solubilization effect, and combined with the chitosan complex coating to achieve slow release antimicrobial effect. The UV-curable system of sugar-based PLA uses sucrose as an initiator to synthesize multi-branched PLA, which has a tensile strength of 25 MPa and a degradation rate of 65% after 6 months of UV-LED curing, constructing a prototype of a fully bio-based printable coating. Nanoemulsions, as efficient nanocarrier systems, can significantly enhance the solubility and bioavailability of active ingredients (e.g., essential oils, hydrophobic drugs, etc.) through a nanoscale particle size. Its high specific surface area promotes contact efficiency with target substances, enhances antimicrobial and antioxidant effects, enhances physical stabilization through surfactant or polysaccharide coatings, inhibits droplet aggregation and sedimentation, maintains structural stability at high temperatures, wide pH ranges, and high ionic strengths, and protects readily degradable components to retard oxidation. Easily prepared at scale by ultrasound or high-pressure homogenization processes, nanoemulsions combine process adaptability and functional tunability [258,259,260]. Table 4 shows representative studies of edible coatings made from nanoemulsions encapsulated with plant essential oils for food preservation applications. These technologies together solve the contradiction of high pollution and low performance of traditional packaging, break through the performance limitations of traditional coatings, and promote food packaging coating technology toward achieving high efficiency, safety and sustainability through material modification and nano-synergistic and green processes, providing systematic solutions for the circular economy.

## 4. Current Challenges and Future Developments

Driven by the current global carbon neutrality target and the circular economy wave, bioplastics, as an environmentally friendly material, are gradually becoming a key solution to replace traditional petroleum-based plastics in the food packaging field [266,267,268]. The core competitiveness of bioplastics in the future will depend on breakthroughs in material science and processing technology. Its processing raw materials are also transitioning from the first generation (corn, sugar cane and other food crops) to the second generation (agricultural waste, straw) and the third generation (algae, microbial synthesis) [269,270,271]. This intergenerational evolution not only alleviates the contradiction of “competing with food for land”, but also significantly improves the efficiency of the circular economy through the resource utilization of lignocellulose and food waste [272,273,274]. Technically, the introduction of innovative processes such as nano-composite and co-mingled modification, has enabled heat resistance, barrier properties and mechanical strength of bioplastics to achieve leapfrog improvement, and some of the properties have even surpassed those of traditional plastics, laying the foundation for the upgrade of functionalization. Intelligent bioplastics, as a material that can sense the freshness of food, will also promote the functionalization of a food packaging upgrade [275,276]. The large-scale utilization of low-cost biomass feedstocks, such as agricultural waste and algae, as well as the optimization of processes such as biosynthesis, are expected to reduce production costs and narrow the price gap with traditional plastics [277,278,279,280].

**Figure 15:**
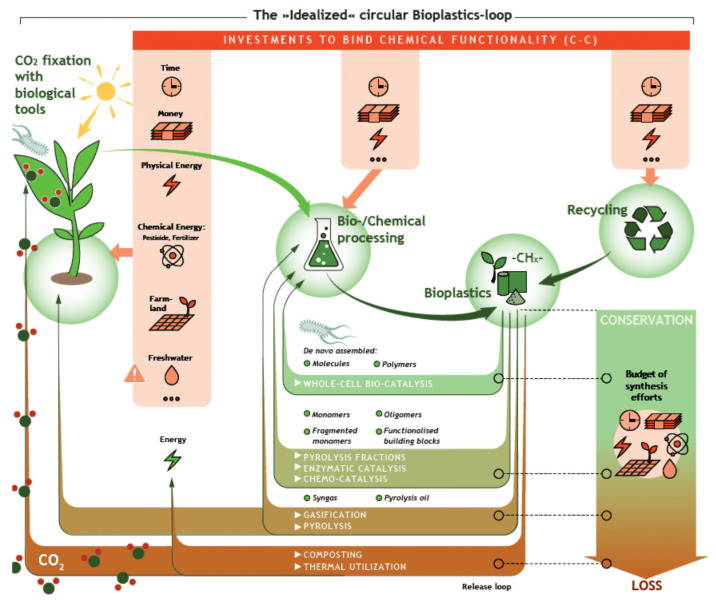
Schematic representation of the vision of an idealized circular Bioplastics loop [294].

As countries continue to ban plastic and implement carbon tariffs, companies may be pushed to turn to biobased materials [281]. Meanwhile, increased consumer preference for “carbon-neutral packaging” and commitments from some brands may accelerate the penetration of bioplastics in the food sector, with global production capacity expected to reach about 5.73 million tons by 2029 [33,282,283]. Currently, bioplastics are still facing problems such as conflicts between raw materials and food security, harsh degradation conditions and lagging recycling infrastructure. Currently, the price of bioplastics is 5–10 times higher than that of traditional plastics, but it is believed that with the optimization of fermentation technology and the expansion of production capacity, the cost of bioplastics is expected to drop to the same level as that of petroleum-based plastics in the future [284,285,286]. In response to these pain points, we are addressing the bioplastics aspect of the problem due to the lack of industrial composting facilities that are compatible with bioplastics through the development and promotion of home compostable certification and the development of materials that can be degraded in natural environments (e.g., marine-degradable PHA), and exploring the conversion of non-food biomass into controllably degradable materials in order to balance environmental friendliness with practicality [287,288,289,290,291,292]. Figure 15 shows a schematic illustration of the vision of an idealized circular bioplastic ring. In the future, the value of bioplastics will not only be “degradable” but will also need to be integrated into life cycle management (LCA). The harmonization of industrial composting facilities and home degradation standards, along with the maturation of chemical recycling technologies (e.g., enzyme depolymerization), will also facilitate the closing of the loop from “bioplastics–organic fertilizers–recycled raw materials” [293,294,295,296]. In the short term, its application may still be dominated by high-performance niche scenarios, while in the long term, it is expected to reshape the packaging industry through the recycling model and become an important pillar of the low-carbon economy [297,298,299]. However, only by breaking through the existing bottlenecks of cost performance and recycling can bioplastics for food packaging truly realize the leap from “environmental alternatives” to “mainstream packaging materials”.

## 5. Summary

This paper summarizes the research progress and application status of bioplastics for food packaging. With the increase in plastic pollution, bioplastics have become a research hotspot in the field of food packaging by virtue of their renewable and degradable characteristics. Biobased biodegradable plastics, with good biocompatibility and degradability, have a wide range of applications in food packaging, and each has its own advantages, such as the thermal stability of PLA, the gas barrier of PHA, the antimicrobial properties of chitosan, and the environmental friendliness of cellulose-based materials. Biobased non-biodegradable Bio-PE and Bio-PET are not biodegradable; however, they are environmentally friendly, have good performance, and are also used in food packaging. Non-biobased biodegradable PBAT, PCL and PBS have good performance and degradability, often through composite modification to optimize performance for food packaging.

People can use plastic processing technologies, including thermoforming, injection molding, extrusion molding and coating technology. Thermoforming is used to produce lunch boxes, which can improve the strength and stability of packaging materials; injection molding can be used to prepare various types of food packaging products, and the performance can be improved through the optimization of processes and raw materials; extrusion molding is mainly used to produce products such as films, which has the advantage of high efficiency and continuous production; and coating technology is used to enhance the barrier, freshness and antimicrobial properties of packaging.

Bioplastics have a bright future in food packaging, but face problems such as conflicts between raw materials and food security, harsh degradation conditions, lagging recycling infrastructure and high costs. In the future, with technological progress, the processing of raw materials will be transformed into non-food biomass, and the performance is expected to exceed that of traditional plastics; smart bioplastics will also promote the upgrade of packaging functions. The bioplastics market size is expected to expand under the impetus of policy and the consumer market.

## Figures and Tables

**Table 1:** Companies that have commercialized PHA production.

Company	PHA Type	Technology	Scale (ton/year)	Websites
PhaBuilder, Beijing, China	All types	*Halomonas* spp. (NGIBa)	1000–10,000	www.phabuilder.com
Medpha, Zhuhai, China	P3HB4HB	*Halomonas* spp. (NGIB)	100	www.medpha.cn
COFCO, Beijing, China	PHB	*Halomonas* spp. (NGIB)	1000	www.cofco.com
Bluepha, Beijing, China	PHBHHx	*Ralstonia eutropha* and NGIB	1000	www.bluepha.com
TianAnBiopolymer, Ningbo, China	PHBV	*R. eutropha*	2000	www.tianan-enmat.com
DanimerScientific, Bainbridge, GA, USA	PHBHHx	*R. eutropha*	10,000	danimerscientific.com
Kaneka, Osaka, Japan	PHBHHx	*R. eutropha*	5000	www.kaneka.be
RWDC, Singapore and Athens, GA, USA	PHBHHx	*R. eutropha*	Unknown	www.rwdc-industries.com
GreenBio, Taizhou, China	P3HB4HB	*Escherichia coli*	10,000	www.greenbio.cn

**Table 2:** Chitosan-based films in different packaging applications.

Active Components	Function	Food	Reference
gelatine	Antioxidant	fruits	[41]
ϵ-polylysine	AntioxidantAntimicrobial	Beef fillet	[116]
α-tocopherol	Antioxidant	Mushroom	[117]
MO	Antioxidant	Cheese	[20]
Butterfly pudding extract	pH indicator	Fish	[118]
Purple tomato	Freshness	Milk and fish	[119]
Tea polyphenols	Freshness	Meat, fruits and vegetables	[120]
TA	UV/Antioxidant	Apple	[121]

**Table 4:** Representative study of edible coatings made from nanoemulsions encapsulated with plant essential oils for food preservation applications.

Essential Oils	Preparation Method of Nanoemulsions	Formula of Coating Solution	Foods	Main Effects	References
Cinnamon	Ultrasonication	Pullulan solution (2 g/100 mL), glycerol (15 g/100 g pullulan polysaccharide) + Tween 80 and 8% of CEO	Strawberries	Pullulan-CEO NE coating remarkably lowered the loss in fruit mass, firmness, and showed the strongest antimicrobial activity against bacteria and molds, respectively.	[261]
Tea tree	Ultrasonication	TTO (1% dissolved in 10 mL of ethanol) + Tween 80 (0.3%) incorporated with LMWCS (low molecular weight chitosan) solution	Fresh cut red bell pepper (FCRBP)	The texture, sensory behavior, and overall quality of FCRBP were maintained for 18 days through controlling the contamination of foodborne pathogenic fungi and bacteria including Salmonella enterica, and Listeria monocytes.	[262]
Curcumin and Orange essential oils	Ultrasonication	The nanoemulsions comprise of 85% aqueous phase, 5% (*w*/*v*) soy protein, and 10% (*v*/*v*), curcumin or orange essential oil	Strawberries	Exhibits the highest EAB and lowest WVP, highly effective bacterial inhibition, excellent freshness retention	[263]
Cinnamon, lemongrass, oregano and citronella	Ultrasonication	Ca-Cas (5% *w*/*v*) and glycerol (2.5% *w*/*v*) + emulsifiers and EOs (cinnamon, lemongrass, oregano, and citronella) with citrus extract and cranberry juice	Carrot	The coating showed a synergistic potential and a higher efficiency to extend the shelf-life of carrots and maintain their quality throughout storage, compared to single treatments.	[264]
Star anise	Ultrasonication	SPI (0.5%, 1%, 1.5% (*w*/*v*)) and lecithin 0.05% (*w*/*w*) + water-soluble polyline (0.067 wt.%) and nisin (0.133% wt.) + 0.4% wt. essential oil and 99.6% wt. water phase + glycerol	Ready-to-eat Yao meat	Samples with NEAC showed the best color, odor and overall acceptance, and the effect of coating with essential oil on sensory acceptability was improved	[265]

## Data Availability

The original contributions presented in this study are included in the article. Further inquiries can be directed to the corresponding author.
